# Metabolites of traditional Chinese medicine targeting PI3K/AKT signaling pathway for hypoglycemic effect in type 2 diabetes

**DOI:** 10.3389/fphar.2024.1373711

**Published:** 2024-05-10

**Authors:** Yuhan Feng, Yan Ren, Xia Zhang, Songqin Yang, Qian Jiao, Qiuhong Li, Wenwen Jiang

**Affiliations:** School of Pharmaceutical Sciences, Guizhou University, Guiyang, China

**Keywords:** metabolites, traditional Chinese medicine, type 2 diabetes mellitus, insulin resistance, PI3K/AKT signaling pathway

## Abstract

Type 2 diabetes mellitus is a chronic metabolic disease characterized by insulin resistance, with high morbidity and mortality worldwide. Due to the tightly intertwined connection between the insulin resistance pathway and the PI3K/AKT signaling pathway, regulating the PI3K/AKT pathway and its associated targets is essential for hypoglycemia and the prevention of type 2 diabetes mellitus. In recent years, metabolites isolated from traditional Chinese medicine has received more attention and acceptance for its superior bioactivity, high safety, and fewer side effects. Meanwhile, numerous *in vivo* and *in vitro* studies have revealed that the metabolites present in traditional Chinese medicine possess better bioactivities in regulating the balance of glucose metabolism, ameliorating insulin resistance, and preventing type 2 diabetes mellitus via the PI3K/AKT signaling pathway. In this article, we reviewed the literature related to the metabolites of traditional Chinese medicine improving IR and possessing therapeutic potential for type 2 diabetes mellitus by targeting the PI3K/AKT signaling pathway, focusing on the hypoglycemic mechanism of the metabolites of traditional Chinese medicine in type 2 diabetes mellitus and elaborating on the significant role of the PI3K/AKT signaling pathway in type 2 diabetes mellitus. In order to provide reference for clinical prevention and treatment of type 2 diabetes mellitus.

## Graphical Abstract

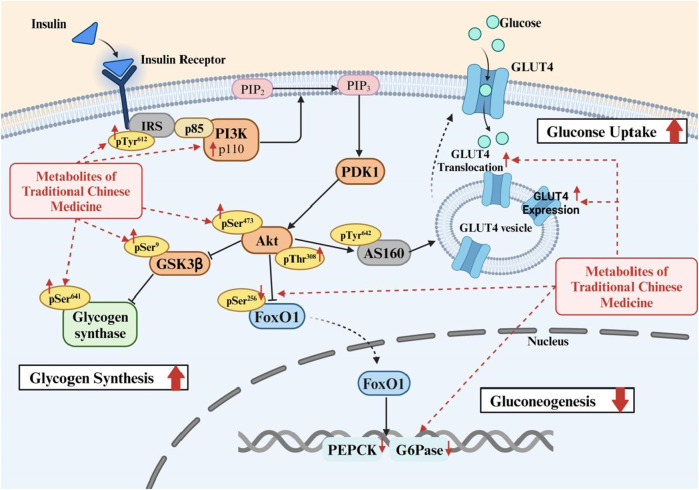

## 1 Introduction

Nowadays, type 2 diabetes (T2DM) is one of the most severe and frequent chronic diseases of the modern era. It has become the third primary non-communicable disease after tumors and cardiovascular diseases, threatening human life and health on a wide scale. According to the statistics released by the International Diabetes Federation, more than 10% of the world’s population became diabetic in 2021 (Sun et al., 2022). Moreover, the prevalence rate is expected to be more than 12% by 2045, which means that more than one in ten people worldwide are suffering from the double blow of diabetes to human health and economic burden, threatening the health of human life and quality of life, of which more than 90% of the patients are type 2 diabetes (Sun et al., 2022). However focusing on hyperglycemia that defines T2DM is mainly secondary to inadequate action of the primary glucose-lowering hormone insulin. Further, understanding insulin resistance and the mechanisms of insulin action is critical for the continued development of effective therapeutic strategies to combat T2DM, which is a major challenge for the medical community (Petersen and Shulman, 2018).

Insulin resistance (IR) is the pathological basis of T2DM and the core part of its pathogenesis, which plays a vital role in both the occurrence and development of T2DM. Generally, IR refers to the inability of insulin-target organs/tissues (e.g., skeletal muscle, adipose tissue, and liver) to produce a normal coordinated glucose-lowering response due to reduced responsiveness and sensitivity to insulin under the influence of a variety of factors, including inhibition of endogenous glucose production, inhibition of lipolysis, cellular uptake of available plasma glucose, and glycogen synthesis, resulting in the onset and worsening of glucose tolerance abnormalities and diabetes mellitus (Petersen and Shulman, 2018).

Clinically, IR is characterized by the inability of insulin to exert an effect proportional to blood concentration to maintain normoglycemia. At the cellular level, IR is defined as the lack of insulin signaling intensity associated with multiple mitogenic cellular functions from downstream receptors to final substrates (Camer et al., 2014). It also implies a corresponding inhibitory effect on the phosphorylation of insulin receptor substrate (IRS) and its triggered cascade of activation of the PI3K/AKT signaling pathway upon binding of insulin to the insulin receptor (Savova et al., 2023). Furthermore, the insulin-mediated PI3K/AKT pathway is crucial for regulating glucose homeostasis in the insulin signaling system, closely related to glucose-lipid metabolism and insulin resistance (Malik et al., 2019). Therefore, improving insulin resistance by modifying the PI3K/AKT pathway is a crucial therapeutic strategy for hypoglycemia in T2DM.

Currently, the primary drugs used in the clinical treatment of T2DM include biguanides, thiazolidinediones, and sulfonylureas (Zhang et al., 2019). Considering the limitations of the existing hypoglycemic drugs that need to be taken for long-term and have plenty of side effects, it is particularly urgent to continuously explore and develop practical, low-toxicity or non-toxic hypoglycemic drugs.

In the theory of traditional Chinese medicine (TCM), diabetes and thirst-quenching disease belong to the same category, and the first record of diabetes was found in the Yellow Emperor’s Classic of Internal Medicine, also known as “Huangdi Neijing” (Zhang et al., 2021). Since ancient times, the threat of diabetes to the life and health of human beings has gradually been growing. Moreover, the research on treating diabetes utilizing traditional Chinese medicine and Chinese medicine approaches has never ceased (Yingrui et al., 2022). From the perspective of TCM, “consuming thirst syndrome” manifests as a deficiency of both Qi and Yin, and the corresponding treatment is to replenish Qi and nourish Yin (Li et al., 2004). Since centuries ago, Chinese people have been decocting Chinese medicine to treat diabetes based on benefiting Qi and nourishing Yin (Liu et al., 2022). For example, traditional Chinese medicine such as astragalus, ginseng, schizandra, and dendrobium are often found in Chinese herbal formulas for the treatment of T2DM, and they are commonly known to nourish yin and replenish qi (Tang et al., 2017; Chu et al., 2019; Shao et al., 2020). Moreover, Tianqi capsule, Jinlida granule, Shenqi jiang tang capsule, Qiyao xiaoke capsule, Shenqi jiang tang granule, which are proprietary Chinese medicines, are used for the treatment of T2DM and its related diseases (Shao et al., 2020). But, TCM is characterized by multiple courses of treatment, slow onset of action, and poor patient adherence (Nurcahyanti et al., 2021). Further, there are hundreds of metabolites in extracts, and the specific metabolites with beneficial effects and their targets of action are unknown (Xiong et al., 2010). Therefore, more and more researchers are deeply interested in the intervention of the TCM metabolites to improve insulin resistance and hyperglycemia. Subsequently, researchers have identified multiple bioactive metabolites isolated from TCM that have positive effects on improving insulin resistance and hypoglycemia, and these bioactive metabolites exhibit a promising therapeutic outlook owing to their biocompatibility and fewer adverse effects (Li et al., 2022). Among the multitude of metabolites present in TCM, the identification of specific active metabolites that can regulate the balance of glucose metabolism and improve insulin resistance is essential for controlling the incidence and progression of T2DM and its complications (Xiong et al., 2020).

Therefore, we conducted a literature search on the keywords “Insulin resistance,” “Type 2 diabetes,” “PI3K/AKT signaling pathway,” “Traditional Chinese medicine” and “Metabolites.” The following inclusion criteria were used in the selection of articles: 1) articles using *in vitro* or/and *in vivo* T2DM or T2DM-related disease models, and modeling methods with a blank control group and a positive control group to determine the success of the modeling; 2) articles in which the study was on a metabolite of TCM were identified as a metabolite reported in the literature or obtained by extraction and isolation from a TCM; 3) articles on the regulation of PI3K/AKT signaling pathway in the context of type 2 diabetes mellitus; 4) articles written in English and published within the last 15 years. Nowadays, researchers are concerned about the advantages and potential of TCM metabolites in the prevention and treatment of chronic diseases, and there are numerous studies showing the positive effects of the metabolites of TCM in improving insulin resistance and hyperglycemia as well as elucidating their hypoglycemic mechanisms, but there is no review summarizing these studies. This review is aimed to provide a comprehensive perspective of the metabolites of TCM improving IR and possessing therapeutic potential for hyperglycemia in T2DM by targeting the PI3K/AKT signaling pathway. Further, it is expected to provide a theoretical basis and reference for future clinical studies.

## 2 PI3K/AKT signaling pathway

Phosphoinositide 3-kinase (PI3K) is a target of the insulin receptor substrate (IRS) and an intracellular phosphoinositol kinase with serine/threonine kinase activity (Xu et al., 2014). Meanwhile, it plays a prominent role in insulin signaling. PI3K can express three types of PI3KⅠ, PI3KⅡ, and PI3KⅢ in human cells (Jahandideh and Wu, 2022). Class I PI3Ks are divided into classes IA and IB PI3Ks (Meng et al., 2021). Heterodimers composed of catalytic subunit P110 and regulatory subunit P85 belong to class IA PI3K, which are involved in insulin signal transduction and have indispensable significance in maintaining glucose homeostasis (Yang et al., 2020).

As a serine/threonine kinase, AKT (protein kinase B) is one of the main effectors of the downstream signal network of PI3K (Huang et al., 2018). AKT is widely expressed in various body tissues, regulating many processes, including metabolism, proliferation, cell survival, growth, and angiogenesis (Liu et al., 2023). Due to its ability to regulate most of the PI3K-mediated metabolic activity of insulin by phosphorylating serine and/or threonine from downstream substrates, including other kinases, transcription factors, and signaling proteins, it is known as the central regulator of insulin action (Shao et al., 2020). Moreover, there are three subtypes of AKT expressed in mammalian cells, namely, AKT1/PKBα, AKT2/PKBβ, and AKT3/PKBγ, and each of these subtypes exerts different physiological effects (Hay, 2011). Among them, AKT1 is widely expressed in various body tissues (Hajiaghaalipour et al., 2015). AKT2 is selectively expressed in insulin-sensitive tissues such as muscle, fat, and liver, and plays a pivotal role in cell growth, proliferation, and glucose homeostasis (Huang et al., 2018). Meanwhile, AKT3 is highly expressed in the brain and testis (Sun et al., 2018). Furthermore, researches have found that all three subtypes of AKT are all expressed in pancreatic β cells (Zheng et al., 2016). Other studies have found that interrupting AKT2 may lead to severe insulin resistance, diabetes, and fat atrophy (Dewanjee et al., 2022). Further, AKT2 gene knockout mice exhibit glucose intolerance and systemic insulin resistance (Alshehade et al., 2022). Among the three subtypes, AKT2 appears to be the primary functional subtype of insulin response, which is closely relevant to IR (Fruman et al., 2017).

PI3K/AKT signaling pathway plays an indispensable role in insulin signal transduction and glucose metabolism regulation and participates in the entire process of T2DM occurrence and development (Lin et al., 2022). PI3K is the leading component of this pathway while AKT is a crucial downstream signal, representing the crucial regulatory node of this pathway (Barone et al., 2021). Insulin is the only hormone in the body to lower blood sugar and its physiological effect begins with the binding of insulin and insulin receptors (Norton et al., 2022). Upon insulin binding to the α-subunit of the insulin receptor, the β-subunit transitions to an activated state, causing its tyrosine residues to self-phosphorylate. Meanwhile, the activated insulin receptor recognizes and binds to the insulin receptor substrate 1 (IRS1), and the phosphorylation and activation of IRS1 can then be recognized and bound by downstream signals (Nurcahyanti et al., 2021). Activated IRS-1 can recognize and bind to PI3K through its regulatory subunit p85 and activate the catalytic activity of p110 and the catalytic subunit of PI3K and catalyze the conversion of phosphatidylinositol 4,5-bisphosphate (PIP2) to phosphatidylinositol 3,4,5-trisphosphate (PIP3) at the cellular membrane (Jahandideh and Wu, 2022). Further, PIP3, when generated and reached a specific concentration, recruits phosohoinositide-dependent kinase 1 (PDK1) and AKT to the vicinity of the plasma membrane and then recruits them through Pleckstrin homologous structures. After PIP3 is generated and reaches a specific concentration, it can recruit PDK1 and AKT to the vicinity of the plasma membrane and bind to it through the Pleckstrin homology domain, resulting in the aggregation of AKT at the plasma membrane and activation of AKT (Fruman et al., 2017). Moreover, the activation process of AKT consists of PDK1 directly or indirectly phosphorylating the Thr308 and Ser473 sites of AKT. Meanwhile, the activated AKT is released from the plasma membrane into the cytoplasm or nucleus to elicit a cascade of responses in the signal transduction pathway (Inam et al., 2018; Alshehade et al., 2022).

Activation of the PI3K/AKT signaling pathway can induce multiple downstream factors, including other kinases, transcription factors, and signal proteins (Bhattamisra et al., 2021). Among the numerous physiological effects that can be generated, T2DM-related effects on glucose metabolism include promoting glucose uptake and glycogen synthesis, and inhibiting gluconeogenesis. AKT can promote the transport of glucose-specific transporters GLUTs to the cell membrane, thereby allowing glucose in the blood to be transported into tissue cells, increasing glucose uptake (Dewanjee et al., 2022). After glucose is transported from extracellular to intracellular tissues, it is phosphorylated in tissues by glucokinase to form glucose-6-phosphate, which continues to undergo conversion to glycolysis or glycogen synthesis (Zhang et al., 2018). Therefore, the PI3K/AKT pathway plays a crucial role in insulin-stimulated glucose transport (Shao et al., 2020).

AKT can inhibit the activity of glycogen synthase kinase-3 (GSK3β) by phosphorylating its Ser9, thereby dephosphorylating and activating glycogen synthase (GS) to regulate insulin signal transduction and increase glycogen synthesis. Glycogen, as a reservoir of glucose, is mainly present in the skeletal muscle and liver of mammals, where it buffers changes in circulating glucose levels by phosphorylating excess glucose and polymerizing it to synthesize glycogen (Norton et al., 2022). Additionally, AKT can reduce the expression of two vital glycogenic enzymes, phosphoenolpyruvate carboxykinase (PEPCK) and glucose 6-phosphatase (G6Pase) by inhibiting the activity of forkhead transcription factor family member (FoxO1) (Wang et al., 2015). Moreover, FoxO1 is located in the nucleus and trans-activates two gluconeogenesis without insulin. Under insulin stimulation, the activated AKT then phosphorylates FoxO1, inducing FoxO1 translocation to the cytoplasm, thereby reducing its transcriptional activity, reducing the expression of its decisive target genes PEPCK and G6Pase, and inhibiting gluconeogenesis to reduce glucose levels (Hajiaghaalipour et al., 2015). Therefore, abnormalities in any PI3K/AKT signaling pathway and downstream factors can affect insulin signal transduction and promote the occurrence of IR and T2DM. On the contrary, the upregulation of PI3K and AKT molecules caused by insulin and other factors can initiate the transmission of the entire PI3K/AKT signal pathway, acting on a variety of substrate receptor molecules such as GLUT-4, GSK3, and FoxO1 through a series of signal transduction, playing a prominent role in increasing glucose uptake, inhibiting liver glycogen synthesis, reducing gluconeogenesis, and improving IR. Therefore, the PI3K/AKT signaling pathway is the primary mechanism for developing insulin resistance.

## 3 Mechanism of PI3K/AKT signaling pathway in hypoglycemic effect

**FIGURE 1:**
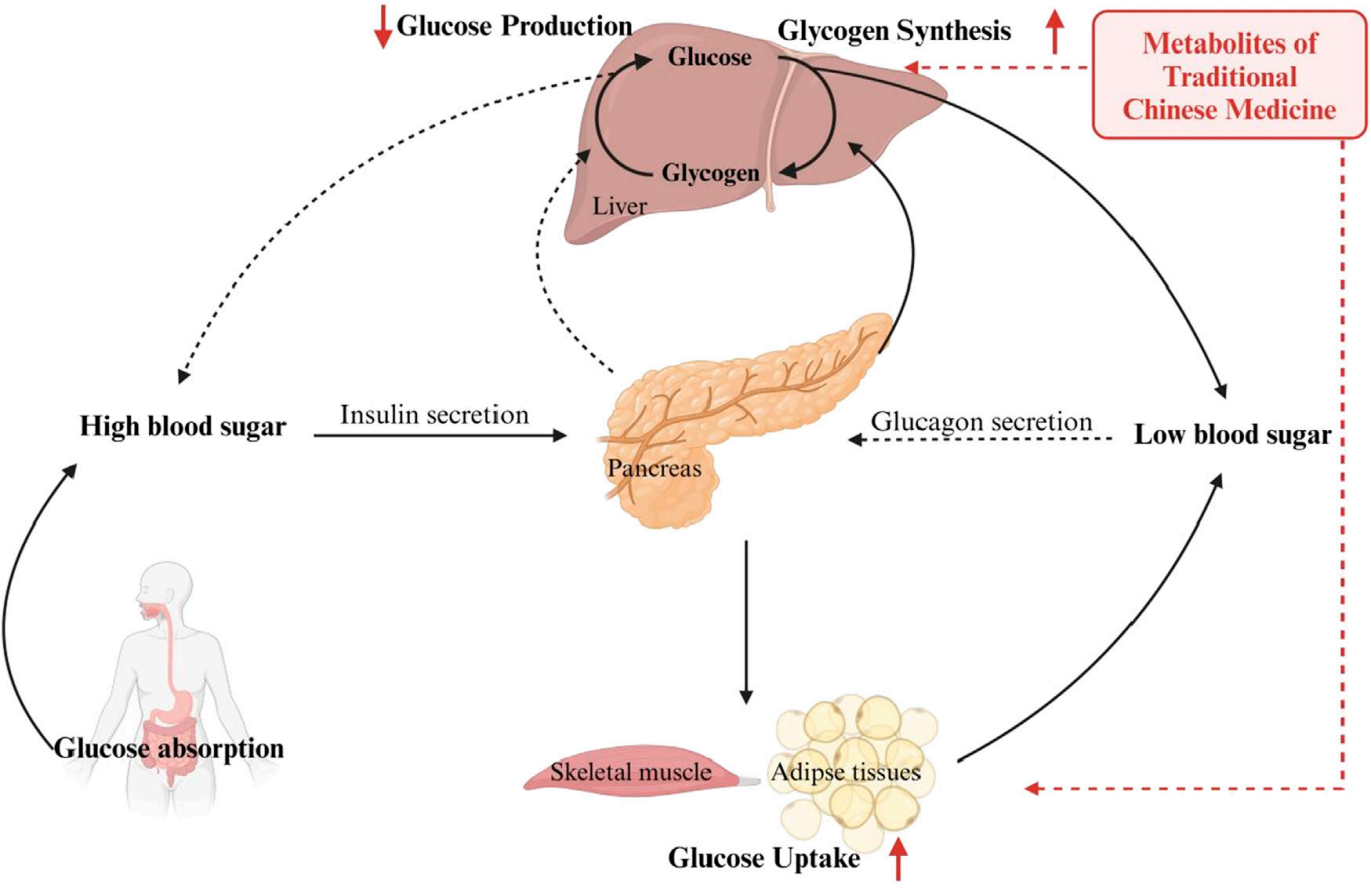
Peripheral control of glucose homeostasis.

The PI3K/AKT signaling pathway is intimately linked to insulin resistance, and any defects in the pathway along downstream molecules may contribute to insulin resistance, in addition, the activated PI3K/AKT pathway is primarily involved in the glucose metabolism function of insulin via three notable routes (Zhang et al., 2019). Peripheral control of glucose homeostasis is shown in Figure 1.

1) Glucose uptake: The cellular uptake of glucose is an essential physiological process associated with glucose homeostasis and glucose translocation from the extracellular space to the cell cytoplasm, and is responsible for fourteen members of the glucose transporter protein family (GLUT) (Yan, 2017). Available evidence from multiple research in the area of diabetes suggests that GLUT4 mediates insulin-dependent glucose uptake and that the PI3K/AKT pathway regulates the movement of GLUT4 between the plasma membrane and intracellular vesicles in an insulin-dependent manner, and thus GLUT4 dysfunction could induce insulin resistance (Chang et al., 2023). Thus, in skeletal muscles and mature adipocytes, the activated PI3K/AKT pathway promotes GLUT4 membrane translocation to uptake glucose for storage or utilization (Savova et al., 2023).
2) Glycogen synthesis: In skeletal muscle and liver, excess glucose is taken up into the cell for storage in the form of synthesized glycogen, thereby increasing the disposal of glucose (Carnagarin et al., 2015). Glycogen synthase kinase-3 (GSK-3), a serine/threonine kinase, is well recognized as a key regulator involved in the regulation of glycogen synthesis and has been suggested as a potential target for the treatment of diabetes (Teli and Gajjar, 2023). Phosphorylation of Ser9 of GSK3β in a PI3K/AKT-dependent manner decreases its activity towards GS, resulting in the promotion of dephosphorylation of glycogen synthase, ultimately leading to an increase in glycogen synthesis to promote glucose metabolism (Seo et al., 2008; Agius, 2015; Beurel et al., 2015).
3) Gluconeogenesis: Glycemic control is achieved by suppressing hepatic gluconeogenesis, which is one of the strategies for the treatment of diabetes (Jitrapakdee, 2012; Nirmalan and Nirmalan, 2023). Gluconeogenesis is largely controlled by the transcriptional regulation of key rate-limiting enzymes, and PEPCK and G6Pase serve as key rate-limiting enzymes, and the regulation of their expression is affected by the phosphorylation of the transcription factor FoxO1 (Onyango, 2022). FoxO1 is activated by dephosphorylation and translocates into the nucleus, leading to increased transcriptional induction of G6Pase and PEPCK as well as hepatic glucose production, whereas when activation of the PI3K/AKT pathway phosphorylates FoxO1, it induces the translocation of FoxO1 to the cytoplasm, thereby decreasing its transcriptional activity, reducing the expression of PEPCK and G6Pase, and inhibiting gluconeogenesis so as to lower blood glucose levels (Jitrapakdee, 2012; Zhang et al., 2018).

## 4 Metabolites of traditional Chinese medicine regulate the PI3K/AKT signaling pathway for hypoglycemic effect

Metabolites isolated from TCM are active substances with various practical activities due to their specific molecular formula and spatial structure (Zhou et al., 2021). Recent studies have clarified that numerous metabolites of TCM addressing complex and comprehensive targets have the potential for hypoglycemic effect in T2DM by regulating the PI3K/AKT signaling pathway (Nurcahyanti et al., 2021). Further, relevant phytochemicals include flavonoids, polyphenols, alkaloids, terpenoids, quinones, saponins and others. These metabolites of TCM may provide promising candidates for improving insulin resistance in the treatment of T2DM, and the effects of the metabolites of TCM on T2DM via the PI3K/AKT signaling pathway are summarized in Table 1.

**TABLE 1:** The effect of metabolites of TCM for hypoglycemic effect in T2DM via the PI3K/AKT signaling pathway.

Type	Name	Study design	*Vivo*/*Vitro*	Dosage of administration	Targets	Mechanism	References
Flavonoids	Baicalein	Male C57BL/6 J mice fed HFD	*in vivo*	400 mg/kg Baicalein for 3 weeks	p-IRS1↑ p-AKT↑ p-AMPKα↑ p-ACC↑	Improving dyslipidemia and insulin resistance	Pu et al. (2012)
		HepG2 cells + glucose + DXMS	*in vitro*	12.5, 25 μM Baicalein for 24 h	p-IRS1/2↑ PI3K↑ p-AKT↑ p-GSK3β↑ GLUT4↑	Promoting glucose consumption and glycogen synthesis	Miao et al. (2023)
		HepG2 cells + glucose + insulin	*in vitro*	1, 10 μM Baicalein for 24 h	p-IRS1↑ p-PI3K↑ p-AKT↑ GLUT2↑ G6Pase↓ PEPCK↓	Promoting glucose uptake and glycolysis, inhibiting gluconeogenesis	Yang et al. (2019)
	Chrysin	Male C57BL/6 J mice fed HFD + STZ	*in vivo*	15, 30 mg/kg Chrysin for 5 weeks	p-AMPKThr172↑ p-IRS1Tyr612↑ p-AKTSer473↑ GSK3βSer9↑ p-GSSer641↓	Modulating glucose and lipid metabolism	Zhou et al. (2021)
		HepG2 cells + glucose + PA	*in vitro*	10, 15 μM chrysin for 24 h			
	Diosmetin	KK-Ay diabetic mice fed HFD	*in vivo*	20, 60 mg/kg Diosmetin for 4 weeks	p-IRS1↑ PI3Kβ↑ PI3Kα↑ p-AKT↑ p-GSK3β↓ GS↑ GLUT4↑ p-AS160↑	Ameliorating glucose metabolism	Gong et al. (2021)
	Tricin	Male C57BL/6 mice	*in vivo*	16, 64, 160 mg/kg Tricin for 7 days	p-IRS1↑ p-PI3KTyr199↑ p-AKTThr308↑ p-AS160Thr642↑	Increasing glucose uptake	Kim et al. (2017)
		C2C12 Myotubes + insulin	*in vitro*	5, 10, 20 µM Tricin for 24 h			
	HM-Chromanone	Male C57BL/KsJ-db/db mice	*in vivo*	30 mg/kg HM-Chromanone for 6 weeks	p-IRS1Tyr612↑ PI3K↑ p-AKTSer473↑ PM-GLUT4↑	Reduceing hyperglycemia and ameliorating dyslipidemia	Yoo et al. (2023)
		L6 cells + PA	*in vitro*	10, 25, 50 µM HM-Chromanone for 24 h	p-IRS1Tyr612↑ p-IRS1Ser307↓ PI3K↑ p-AKT↑ p-AS160↑ PM-GLUT4↑ p-GSK3α/β↑ p-GS↓	Stimulating glucose uptake and glycogen synthesis, and improving insulin resistance	Park et al. (2019)
		L6 cells + PA	*in vitro*	10, 20 µM HM-Chromanone for 24 h	p-IRS1Tyr612↑ PI3K↑ p-AKT↑ p-AMPK↑ p-AS160↑ p-GSK3α/β↑ p-GS↓ PM-GLUT4↑	Stimulating glucose uptake and glycogen synthesis	Park et al. (2021)
		HepG2 cells +33 mM glucose	*in vitro*	10, 20, 50 µM HM-Chromanone for 24 h	p-IRS1Tyr612↑ p-IRS1Ser307↓ p-AKT↑ GSK3βSer9↑ p-GSSer641↓ G6Pase↓ PEPCK↓	Suppressing glucose production and stimulating glycogen synthesis	Park and Han, (2022)
		HepG2 cells + insulin	*in vitro*	15, 30, 60 µM HM-Chromanone for 24 h	p-IRS1 Tyr612↑ p-IRS1 Ser307↓ PI3K↑ p-AKT↑ p-FoxO1↑ PEPCK↓ G6Pase↓	Suppressing hepatic glucose production	Park and Han, (2022)
		3T3-L1 adipocytes + DXMS + insulin	*in vitro*	10, 20 µM HM-Chromanone for 24 h	p-IRS1↑ PI3K↑ p-AKT↑ p-AMPK↑ p-ACC↑ PM-GLUT4↑	Enhancing glucose uptake and insulin sensitivity	Park et al. (2019)
	Puerarin	Male Wistar rats fed HFD + STZ	*in vivo*	300 mg/kg puerarin for 4 weeks	p-AKT↑ PI3K↑ p-FoxO1↑ G6Pase↓ PEPCK↓	Suppressing gluconeogenesis	Liu et al. (2021)
		HepG2 cells + PA	*in vitro*	10, 100, 1000 μM puerarin			
		Male C57BL/6 mice + STZ	*in vivo*	100 mg/kg puerarin for 4 days	p-AKT↑ BCL2↑	Protecting pancreatic b-cell function and survival via direct effects on b-cells	Li et al. (2014)
		MIN6 cells + CoCl2	*in vitro*	0.1, 1, 10 mM puerarin for 8 h			
		HepG2 cells + insulin	*in vitro*	1, 10, 100 μM puerarin	p-AKT1↑ p-GSK-3β↑	improving glucose and lipid metabolism disorders	Liu et al. (2021)
	α-Methyl artoflavanocoumarin (MAFC)	HepG2 cells + insulin	*in vitro*	12.5, 25, 50 µM MAFC for 24 h	PTP1B↓ p-IRS1Ser307↓ p-PI3KTyr508↑ p-AKTSer473↑ p-ERK1Tyr204↑	Increasing glucose uptake	Jung et al. (2017)
	Loureirin B	Male C57BL/6 J mice fed HFD + STZ	*in vivo*	45 mg/kg Loureirin B for 4 weeks	IRS1↑ PI3K↑ p-AKT↑ FoxO1↓ PEPCK↓ GLUT4↑	Increasing insulin sensitivity, and regulating glucose uptake and production	Ding et al. (2021)
		HepG2 cells + glucose + DXMS	*in vitro*	0.1, 1, 10 μM Loureirin B			
	Fisetin	Male C57BL/6 mice fed HFD	*in vivo*	20, 40 mg/kg Fisetin for 16 weeks	p-IRS1↑ p-AKT↑ p-GSK3β↑ p-FoxO1↑	Improving insulin resistance and inflammatory response	Ge et al. (2019)
		HepG2 cells + insulin	*in vitro*	25, 50 μM Fisetin for 24 h	p-IRS1↑ p-AKT↑	Improving hepatic insulin resistance	Li et al. (2023)
	Kaempferol	Male C57BL/6 J mice fed HFD	*in vivo*	50 mg/kg Kaempferol for 6 weeks	p-AKT↑	Ameliorating hepatic gluconeogenesis	Alkhalidy et al. (2018)
		Male C57BL/6 J mice fed HFD	*in vivo*	50 mg/kg Kaempferol for 5 days	p-AKT↑ p-AMPK↑	Improving glucose uptake	Moore et al. (2023)
		Primary human SkM cells	*in vitro*	10 μM Kaempferol for 24 h			
		HepG2 cells	*in vitro*	0.1, 1, 10 μM Kaempferol for 24 h	p-AKT↑ p-GSK3β↑	Improving glucose consumption	Fang et al. (2021)
	Quercetin	Male Wistar rats + STZ	*in vivo*	50 mg/kg Quercetin for 2 months	p-IRS1↑ p-PI3K↑ p-AKT1↑ GLUT4↑	Improving glucose homeostasis in the brain	Sandeep and Nandhini, (2017)
		Male C57BL/6 J mice fed HFD	*in vivo*	50 mg/kg Quercetin for 10 weeks	p-IRS1↑ p-AKT1↑ GLUT4↑ p-FoxO1↑ PEPCK↓ G6Pase↓	Suppressing gluconeogenesis	Liu et al. (2022)
		HepG2 cells + PA	*in vitro*	10 μM Quercetin for 24 h			
		HepG2 cells + PA	*in vitro*	5, 10 μM Quercetin for 24 h	p-IRS2↑ PI3Kp85↑ p-AKT1↑ p-FoxO1↑	Improving hepatic insulin resistance	Cheng et al. (2021)
	Apigenin	HepG2 cells + glucose + DXMS	*in vitro*	6.25, 12.5 μM Apigenin for 16 h	IRS1/2↑ PI3K↑ p-AKT↑ p-GSK3β↑ GLUT4↑	Increasing insulin sensitivity and glucose uptake	Miao et al. (2023)
	Poncirin	C2C12 cells + insulin	*in vitro*	5, 1o μM Poncirin for 16 h	p-IRS1↑ p-PI3K↑ p-AKT1↑ p-GSK3β↑ GLUT4↑	Improving insulin sensitivity and suppressing glycation-induced protein oxidation	Yousof Ali et al. (2020)
	Naringenin	Male Wistar rats + STZ	*in vivo*	50 mg/kg Naringenin for 2 months	p-IRS1↑ p-PI3K↑ p-AKT1↑ GLUT4↑	Improving glucose homeostasis in the brain	Sandeep and Nandhini, (2017)
Polyphenols	Resveratrol	Male Wistar rats fed standard rodent diet + STZ	*in vivo*	10 mg/kg Resveratrol for 4 weeks	insulin Rβ↑ IRS-1↑ eNOS↑ PI3K↑ p-AKT↑	Improving both hepatic inflammation and insulin resistance	Sadi et al. (2015)
		Male C57BL/6N mice fed HFD	*in vivo*	30 mg/kg Resveratrol for 2 weeks	p-IRS-1↑ p-PI3K↑ p-AKT↑ p-PDK1↑ p-GSK-3↑	Restored the phosphorylation levels of proteins involved in the insulin signaling pathway	Hong et al. (2014)
		Male Wistar rats + STZ	*in vivo*	0.05, 0.1, 0.5, 3.0, 6.0, 10.0 mg/kg Resveratrol for 7 days	p-AKTSer473↑ GLUT4↑ PEPCK↓	Increasing insulin secretion and enhancing glucose uptake	Chi et al. (2007)
		Male KKAy mice	*in vivo*	2, 4 g/kg Resveratrol for 12 weeks	p-AMPKα↑ p-IRS1↑ p-AKT↑ Sirt1↑	Improving the insulin sensitivity	Chen et al. (2012)
		Male Sprague-Dawley rats fed HCF	*in vivo*	1 mg/kg Resveratrol for 15 days	p-InsR↑ p-AKT↑ GLUT4↑	Enhancing muscular glucose uptake	Deng et al. (2008)
		Male C57BL/6J mice fed HFD	*in vivo*	100 mg/kg Resveratrol for 6 weeks	p-PI3K↑ p-AKT↑ FoxO1↓ G6Pase↓	Reducing the Glucose Concentration and inhibiting glycoisogen	Shu et al. (2020)
		HepG2 cells + PA	*in vitro*	25 μM Resveratrol for 24 h			
		Male ob/ob mice fed HFD	*in vivo*	10 mg/kg Resveratrol for 10 weeks	PI3K↑ p-AKT↑ p-FoxO1↑ PEPCK↓ G6Pase↓ Sirt1↑ PGC-1α↑	Enhancing glucose production and restraining dapagliflozin-induced renal gluconeogenesis	Sun et al. (2021)
		HK-2 cells	*in vitro*	10 μM Resveratrol for 12 h			
	Pterostilbene	Male Sprague-Dawley rats fed HFD + STZ	*in vivo*	20, 40, 80 mg/kg Pterostilbene for 8 weeks	PPARγ↑ PI3K↑ p-AKT↑ GLUT4↑ IRS-1↑	Controling serum glucose, improving insulin lipid profile and insulin sensitivity	Sun et al. (2019)
		HepG2 cells + PA	*in vitro*	5, 10 μM Pterostilbene for 24 h	p-IRS-1ser307↑ p-AKTser473↑ p-GSK3βser9↑ p-FoxO1↑ PEPCK↓ G6Pase↓	Reducing lipid accumulation and alleviating inflammatory response	Malik et al. (2019)
	Curcumin	Male C57BL/6J mice fed HFS	*in vivo*	4 g/kg curcumim for 16 weeks	PI3Kp110/p85↑ p-AKT↑	Improving insulin clearance, mediating the insulin pathway signaling	Kim et al. (2019)
		Male Sprague-Dawley rats fed HFD + STZ	*in vivo*	100, 300 mg/kg curcumim for 8 weeks	p-PI3K↑ p-AKT↑	Improving liver function and ameliorating the tissue structure of the liver and pancreas	Xia et al. (2020)
		HepG2 cells +50 mM D-glucose	*in vitro*	10 μM curcumim for 24 h	p-AKT↑ p-PI3K↑ p-GSK3β↑	Improving insulin sensitivity, enhancing glucose uptake	Li et al. (2020)
		MIN6 β-cells + PA	*in vitro*	10 μM curcumim for 1 h	p-AKT↑ p-FoxO1↑	Improving glucose-induced insulin secretory function	Hao et al. (2015)
	Gallic acid	Male Sprague-Dawley rats fed HFD	*in vivo*	10, 30 mg/kg Gallic acid for 4 weeks	IRS-1↑ PI3K↑ AKT↑ GLUT-2↑	Improving glucose uptake and decreasing hyperglycemia	Huang et al. (2016)
		Male Wistar rats fed HFD + STZ	*in vivo*	20 mg/kg Gallic acid for 10 days	PPARγ↑ PI3K↑ p-AKT↑ GLUT4↑	Enhancing insulin dependent glucose uptake and improving hyperlipemia	Gandhi et al. (2014)
		HepG2 cells + FFA	*in vitro*	50 μM Gallic acid for 24 h	p-IRS-1↑ p-PI3K↑ p-AKT↑ p-FoxO1↑	Increasing glucose consumption	Lee and Lee, (2021)
Alkaloids	Tetramethylpyrazine	Male Wistar rats fed HFD + STZ	*in vivo*	100, 150, 200 mg/kg TMP for 28 days	p-PI3Kp85↑ p-AKT↑ GLUT4↑	Reducing insulin resistance	Rai et al. (2019)
	Hirsutine	Male C57BL/6J mice fed HFD	*in vivo*	5, 10, 20 mg/kg Hirsutine for 8 weeks	p-AKT↑ p-PDK1↑ p-GSK3β↑ p-AMPK↑ G6Pase↓ PEPCK↓ PGC-1α↓ FoxO1↓	Enhancing glucose consumption, glycogen synthesis, and suppressing gluconeogenesis	Hu et al. (2022)
		HepG2 cells + D-glucose + insulin	*in vitro*	0.01, 0.1, 1 μM Hirsutine for 24 h			
		H9c2 cells + D-glucose + insulin	*in vitro*				
	1-Deoxynojirimycin	Male ob/ob mice	*in vivo*	40, 80 mg/kg 1-Deoxynojirimycin for 4 weeks	p-PI3Kp85↑ p-AKTser473↑ p-IRS1tyr612↑ p-IRβtyr1361↑ GLUT4↑	Improving insulin sensitivity and enhancing glucose uptake	Liu et al. (2015)
		Male ob/ob mice	*in vivo*	40 mg/kg 1-Deoxynojirimycin for 35 days	PPARγ↑ PGC-1α↑ GLUT4↑ IRS-1↑ p-PI3K↑ p-AKT↑ p-GSK3β↑ p-GS↑	Enhancing glucose consumption, glycogen synthesis, and suppressing gluconeogenesis	Kang et al. (2022)
		3T3-L1 adipocytes + DXMS + insulin	*in vitro*	0.1, 0.5, 1, 5, 10 μM 1-Deoxynojirimycin for 24 h	IR↑ IRS-1↑ PI3K↑ AKT↑ AMPK↑ GLUT4↑	Enhancing glucose uptake	Li et al. (2019)
Terpenoids	Mogroside V	Male Wistar rats fed HFD + STZ	*in vivo*	30, 75, 150 mg/kg Mogroside V for 5 weeks	IRS-1↑ PI3KP110/P85↑ p-AKT↑ GLUT2↑ GS↑ p-GSK3β↑	Improving insulin sensitivity, glucose homeostasis and liver damage	Liu et al. (2019)
		HepG2 cells + PA	*in vitro*	1, 5, 10 μM Mogroside V for 24 h			
	Siamenoside I	HepG2 cells + PA	*in vitro*	1, 5, 10 μM Siamenoside I for 24 h	IRS-1↑ PI3KP110/P85↑ p-AKT↑ GLUT2↑ GS↑ p-GSK3β↑	Improving insulin sensitivity, glucose homeostasis and liver damage	Liu et al. (2019)
	Mogroside III	HepG2 cells + PA	*in vitro*	1, 5, 10 μM MogrosideIII for 24 h	IRS-1↑ PI3KP110/P85↑ p-AKT↑ GLUT2↑ GS↑ p-GSK3β↑	Improving insulin sensitivity, glucose homeostasis and liver damage	Liu et al. (2019)
	Mogroside IV	HepG2 cells + PA	*in vitro*	1, 5, 10 μM Mogroside IV for 24 h	IRS-1↑ PI3KP110/P85↑ p-AKT↑ GLUT2↑ GS↑ p-GSK3β↑	Improving insulin sensitivity, glucose homeostasis and liver damage	Liu et al. (2019)
	Catalpol	Male C57BL/6N mice fed HFD + STZ	*in vivo*	100, 200 mg/kg Catalpol for 4 weeks	IRS-1↑ PI3KR1↑ AKT2↑ AMPK↑ p-AMPK↑ GLUT4↑ PGC-1α↑ SIRT1↑ PPAR-γ↑	Improving insulin sensitivity and mitochondrial respiration	Yap et al. (2020)
		Male db/db mice	*in vivo*	200 mg/kg Catalpol for 8 weeks	p-IRS1Ser307↑ PI3K↑ p-AKTSer473↑ GLUT4↑	Improving insulin sensitivity, and enhancing myogenesis ans glucose uptake	Xu et al. (2018)
		C2C12 cells +50 mM D-glucose	*in vitro*	10, 30, 100 μM Catalpol for 24 h			
		C57BL/6J mice fed HFD + STZ	*in vivo*	100, 200 mg/kg Catalpol for 4 weeks	p-IRS1Ser307↑ p-AKTSer473↑ p-GSK3ser9β↑ G6Pase↓ PEPCK↓ p-GSser641↓ p-FoxO1ser256↑	Ameliorating hepatic insulin resistance	Yan et al. (2018)
		HepG2 cells +18 mM glucosamine	*in vitro*	20, 40, 80 μM Catalpol for 24 h			
	Oleanolic Acid	Male db/db mice	*in vivo*	250 mg/kg Oleanolic Acid for 28 days	p-AKT↑ p-PI3K↑ p-AMPK↑ p-ACC↑ G6pase↓ PEPCK1↓ GLUT2↓	Reducing gluconeogenesis, glycogenolysis and hepatic glucose production	Wang et al. (2015)
		Male Wistar rats fed fructose	*in vivo*	5, 25 mg/kg Oleanolic Acid for 10 weeks	p-IRS-1↑ PI3K↑ p-AKT↑	Attenuating adipose tissue insulin resistance	Li et al. (2014)
		C57BL/6J mice fed HFD + STZ	*in vivo*	100 mg/kg Oleanolic Acid for 2 weeks	p-AKTSer473↑ p-FoxO1ser256↑ G6Pase↓ PEPCK↓	Improving glucose homeostasis and reducing gluconeogenesis	Zeng et al. (2012)
	Asiatic Acid	Male Wistar rats + STZ	*in vivo*	20 mg/kg Asiatic Acid for 45 days	IR↑ IRS-1/2↑ PI3K↑ AKT↑ GLUT4↑	Increasing insulin secretion and glucose uptake into skeletal muscle	Ramachandran and Saravanan, (2013)
		Male db/db mice fed HFD	*in vivo*	50 mg/kg Asiatic Acid for 4 weeks	IRS-1↑ PI3K↑ AKT1↑ GSK-3β↓ G6pase↓	Improving glycogen synthesis	Sun et al. (2017)
	Glycyrrhetinic acid	HepG2 cells + PA	*in vitro*	20, 35, 50 μM Glycyrrhetinic acid for 24 h	PI3K↑ p-AKT↑ GSK3β↑	Regulating the insulin resistance	Wang et al. (2023)
		HepG2 cells + insulin/FFA	*in vitro*	5, 10 μM Glycyrrhetinic acid for 24 h	p-IRS1↓ p-AKT↑ p-GSK3β↑ GLUT4↑	Improving glucose uptake and reversing insulin resistance	Zhang et al. (2019)
	Maslinic acid	Preadipocytes	*in vitro*	0.5, 1 μM Maslinic acid for 24 h	PI3K↑ AKT↑	Inhibiting adipocyte differentiation and lipid accumulation	Savova et al. (2021)
		HepG2 cells	*in vitro*	0.1, 1, 10 μM Maslinic acid for 24 h	p-AKT↑ GSK3β↑	Modulating glycogen metabolism	Liu et al. (2014)
Quinones	Aloin	Male mice fed HFSD + STZ	*in vivo*	90 mg/kg Aloin for 4 weeks	IRS1↑ PI3K↑ AKT↑ JNK↑	Enhancing glucose tolerance and glucose consumption	Zhong et al. (2022)
		HepG2 cells + DEX	*in vitro*	1, 10, 50, 100, 200 μM Aloin for 24 h			
	Embelin	Male Wistar rats fed HFD + STZ	*in vivo*	50 mg/kg Embelin for 30 days	PPARγ↑ PI3K↑ p-AKT↑ GLUT4↑	Improving insulin sensitivity, protecting β-cell from damage and maintaining glucose homeostasis	Gandhi et al. (2013)
	Emodin	KK-Ay diabetic mice fed HFD	*in vivo*	12.5, 50 mg/kg Emodin for 8 weeks	p-IRS1↑ p-PI3K↑ p-AKT↑ GLUT2↑ GLUT4↑ PPARγ↑	Enhancing insulin sensitivity and resistance	Xuezheng et al. (2018)
Saponins	Astragaloside IV	3T3-L1 adipocytes + PA + glucose	*in vitro*	10, 50, 100, 200 μM Astragaloside IV for 24 h	PI3K↑ p-AKT↑ GLUT4↑	Improving insulin resistance and inflammation in adipocytes	Zhang et al. (2022)
	Ginsenoside Rb2	DIO mice	*in vivo*	50 mg/kg Ginsenoside Rb2 for 10 days	IRβ↑ IRS1↑ PI3Kp85↑ p-AKTser473↑	Improving insulin sensitivity and reducing fat mass	Dai et al. (2018)
		3T3-L1 adipocytes + DXMS + insulin	*in vitro*	25 μM Ginsenoside Rb2 for 30 min			
	Ginsenoside Rg5	Male db/db mice	*in vivo*	90 mg/kg Ginsenoside Rg5 for 8 weeks	p-IRS1tyr↑ p-IRS1ser↓ p-PI3K↑ p-AKT↑ GSK-3β↑ GS↓	Promoting glycogen synthesis, improving glycolipid metabolism and insulin secretion	Wei et al. (2020)
Others	Beta-sitosterol	Adult Male Albino rats fed HFD	*in vivo*	20 mg/kg Beta-sitosterol for 30 days	IR↑ p-IRS1tyr632↑ p-IRS1ser632↓ p-AKTser473↑ p-AKT thr308↑ GLUT4↑	Improving insulin resistance	Babu et al. (2020)
		L6 cells + glucose	*in vitro*	1 μg/mL, 100 ng/mL Beta-sitosterol for 6, 8, 12 h	p-IRS-1↑ PI3Kp85↑ p-AKT↑ PKC↑ GLUT4↑	Stimulating glucose transport	Sujatha et al. (2010)
		Preadipocytes + DXMS + insulin	*in vitro*	0.1, 1, 10, 100, 1,000, 10,000 μM Beta-sitosterol for 24 h	PI3K↑ AKT↑ GLUT4↑	Regulating glucose uptake, adipogenesis, and lipolysis in adipocytes	Chai et al. (2011)
	Taurine	Male SD rats fed HFD + STZ	*in vivo*	400, 600 mg/mL Taurine for 7 weeks	PI3K↑ AKT↑ GLUT4↑	Stimulating glucose consumption and ameliorating oxidative stress	Chen et al. (2021)
		HepG2 cells + PA	*in vitro*	10, 100, 500 μg/mL Taurine for 24 h			
	1,7-Diphenyl-4E-en-3-heptanone (DPH5)	HepG2 cells + glucose	*in vitro*	10, 20, 40 μM DPH5 for 24 h	p-PI3Kp85↑ p-AKT↑ GLUT4↑ p-GSK3β↑ GCK↑ PK↑ PEPCK↓ G6Pase↓	Promoting glucose uptake and glucose consumption, regulating glucose metabolism and enhancing insulin sensitivity	Zhang et al. (2022)
	(R)-5-hydroxy-1,7-diphenyl-3-heptanone (DPHC)	Male C57BL/KsJ db/db mice	*in vivo*	80, 140 mg/mL DPHC for 8 weeks	IRS1↑ p-PI3K↑ p-AKT↑ GLUT4↑	Regulating blood glucose level and glucose tolerance, improving glucose metabolism	Zhang et al. (2021)
		HepG2 cells + glucose	*in vitro*	10, 20, 40 μM DPHC for 24 h			
	Esculin	Male C57BL/6J mice fed HFD	*in vivo*	40, 80 mg/kg Esculin for 4 weeks	IRS1↑ p-PI3K↑ p-AKT↑ GLUT4↑	Improving adipose tissue remodeling and increasing glucose uptake	Yang et al. (2024)
		3T3-L1 adipocytes + PA	*in vitro*	50, 100 μM Esculin for 24 h			
		Male ICR mice + DXMS	*in vivo*	40 mg/kg Esculin for 21 days	p-AKT↑ p-AMPK↑ GLUT4↑	Promoting glucose uptake and improving insulin resistance	Mo et al. (2019)
		C2C12 Myotubes + DXMS	*in vitro*	25, 50, 100 μM Esculin for 24 h			
		Male ICR mice + STZ	*in vivo*	200 mg/kg Esculin for 14 days	IR↑ p-AKT↑ p-GSK3β↑	Increasing glucose uptake and improving insulin sensitivity	Kang et al. (2014)
		C2C12 Myotubes + insulin	*in vitro*	50 μM Esculin for 24 h			

### 4.1 Flavonoids

**FIGURE 2:**
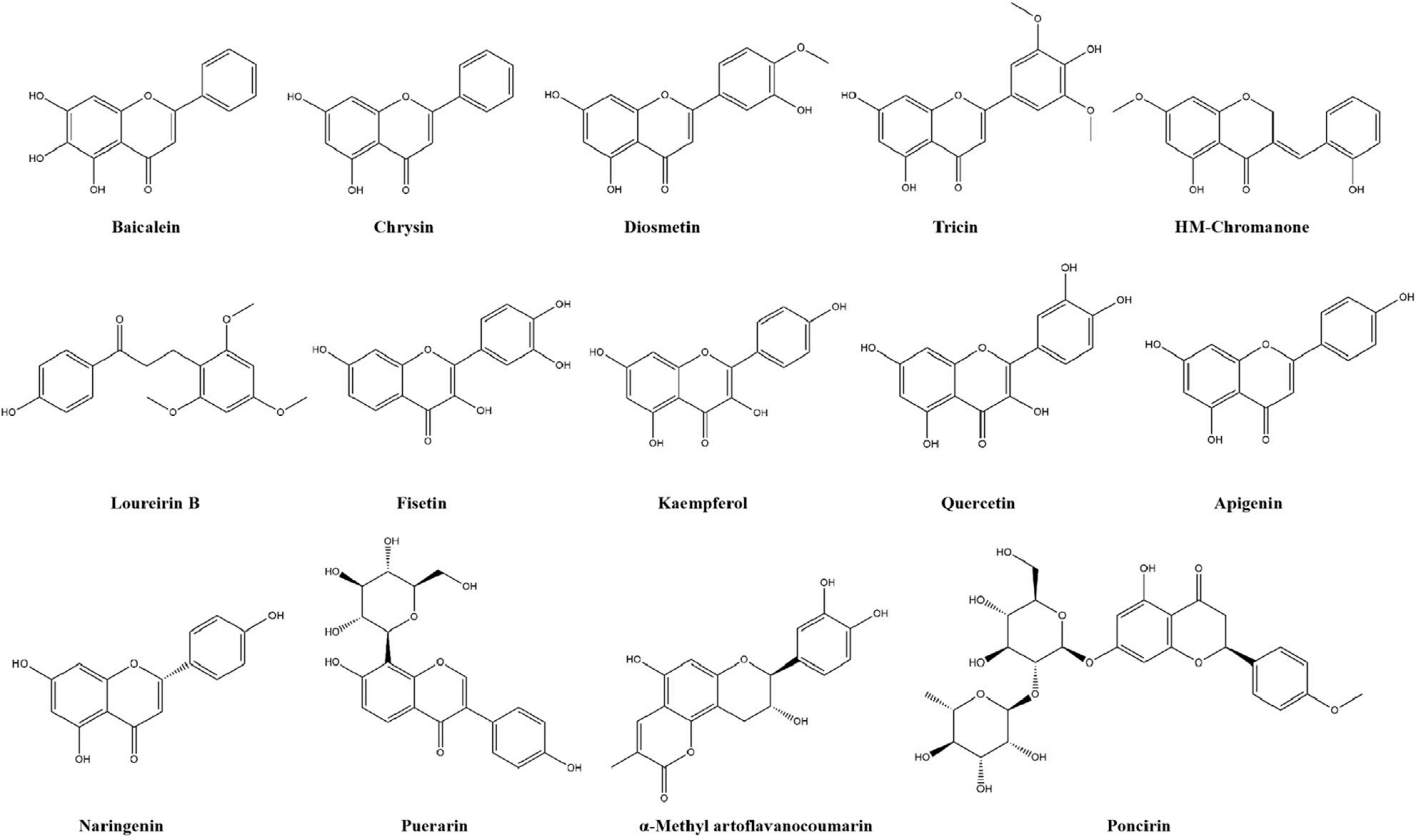
The chemical structures of fourteen flavonoids.

Being commonly found in Chinese herbal medicine, flavonoids are a group of ubiquitous compounds of in nature that have proven to have medicinal value (Sok Yen et al., 2021). Due to their wide range of beneficial activities, such as antioxidant, anti-inflammatory, anti-viral, anti-atherosclerotic, anti-diabetic and anti-tumor, flavonoids have great potential in clinical application and clinical development (Zhou et al., 2023). In recent years, flavonoids extracted from dietary sources and medicinal plants have been widely used in treating and preventing various diseases, and the hypoglycemic potential activities of flavonoids are being explored (Ahad et al., 2014). Moreover, various *in vitro* and *in vivo* experiments have demonstrated the efficacy of flavonoids in improving insulin resistance and preventing T2DM (Zhou et al., 2023), such as baicalein, chrysin, diosmetin, tricin, HM-chromanone, puerarin, α-Methyl artoflavanocoumarin, loureirin B, fisetin, kaempferol, quercetin, apigenin, poncirin and naringenin. The chemical structures of fourteen flavonoids are shown in Figure 2.

#### 4.1.1 Baicalein

Baicalein (5, 6, 7-trihydroxyflavone), one of the representative active metabolites of the medicinal plant *Scutellaria baicalensis Georgi* (known as huáng qín), is a naturally occurring hypoglycemic agent that can directly promote insulin secretion and preserve pancreatic islet mass (Fu et al., 2014). Besides, orally given baicalein (400 mg/kg/day) to C57BL/6 mice induced by a high-fat diet, the disorders of dyslipidemia, fatty liver, diabetes and insulin resistance in mice were effectively normalized after oral medication and all of these improvements were mediated by inhibition of the MAPKs pathway and activation of the IRS1/PI3K/AKT pathway involving multiple intracellular signaling pathways (Pu et al., 2012). Meanwhile, baicalein can also promote glucose consumption and glycogen synthesis and inhibit gluconeogenesis to improve glucose metabolism and the mechanism of the anti-diabetic effect in IR-induced HepG2 cells related to activation of IRS1/PI3K/AKT signaling pathways and the expression of proteins downstream of the pathway (Yang et al., 2019; Miao et al., 2023). The above results show that baicalein might have a promising potential in preventing and treating diabetes and need more exploration in clinical application.

#### 4.1.2 Chrysin

Chrysin (5, 7-di-OH-flavone), a flavone, is a promising phytochemical discovered in a variety of TCM which has been reported emphasizing benefits in numerous metabolic malfunctions such as anti-diabetic effects, anti-cancer and anti-inflammatory role (Samarghandian et al., 2017; Naz et al., 2019). Depending on its adjuvant therapy effects for glucose and lipid metabolism disorders such as IR, oxidative stress, inflammation, and liver injury in both IR-HepG2 cells and HFD/STZ-induced C57BL/6J mice, they found that Chrysin intervention could modify glycogen synthesis and fatty acid oxidation and suppress gluconeogenesis and fatty acid synthesis by regulating the AMPK/PI3K/AKT signaling pathway (Zhou et al., 2021).

#### 4.1.3 Diosmetin

Diosmetin (3′, 5, 7-trihydroxy-4′-methoxyflavone), a naturally occurring flavonoid, has a superior diabetic alleviating effect due to its targeting of α-glucosidase and the PTP-1B signaling pathways (Chen et al., 2019). The treatment of mice with low and high doses of diosmetin remarkably ameliorated glucose metabolism in KK-Ay diabetic mice and regulated the expression of glucose metabolism and insulin resistance related signaling proteins in the liver and skeletal muscle. Hence, they suggested that it ameliorated glucose metabolism and insulin resistance via up-regulating IRS/PI3K/AKT signaling pathway to promote glycogen synthesis and GLUT4 translocation (Gong et al., 2021).

#### 4.1.4 Tricin

As a cereal flavone, Tricin (5, 7, 4′-trihydroxy-3′, 5′-dimethoxyflavone) is widely distributed in the husks of various cereal crops and multiple TCM. tricin possesses an anti-adipogeneic effect, which was reported and suggested for the first time that tricin exhibits a significant inhibitory activity toward adipogenesis and lipogenesis by blocking the AKT/mTOR/S6K signaling pathway (Lee et al., 2015; Lee et al., 2016). However, in a dissimilar way, tricin could enhance GLUT4 translocation and glucose uptake by activating the insulin-dependent PI3K/AKT/AS160 signaling pathway in C2C12 myotubes and the oral administration of Tricin significantly lowered blood glucose levels in glucose-loaded C57BL/6 mice (Kim et al., 2017). These findings indicate that tricin has promising prospects to act as a functional agent for glycemic control.

#### 4.1.5 HM-chromanone

(E)-5-hydroxy-7-methoxy-3-(2′-hydroxybenzyl)-4-chromanone (HM-chromanone), a sapanin homoisoflavonoid that derived from *Portulaca oleracea* L., has an extensively potential in promoting insulin secretion and anti-diabetes effect of a substance isolated from TCM. *In vivo*, HM-chromanone can reduce hyperglycemia and ameliorate dyslipidemia in C57BL/Ksj-db/db mice, the effect of 30 mg/kg HM-chromanone for 6 weeks on levels of HbA1c, plasma insulin, HOMA-IR and serum lipid significantly normalized. Furthermore, the ability of HM-chromanone supplementation to promote the activation of insulin signaling pathways and lead to glucose uptake into skeletal muscle cells was also clarified (Yoo et al., 2023). *In vitro*, HM-Chromanone was found to improve glucose uptake, glycogen synthesis and other cellular functions related to glucose metabolism in L6 skeletal muscle cells, 3T3-L1 adipocytes and HepG2 cells by activating the PI3K/AKT signaling pathway or acting in conjunction with the AMPK signaling pathway (Park et al., 2019; Park et al., 2021; Park and Han, 2022; Park and Han, 2022). Therefore, it shows a more positive effect in all three primary target insulin groups to improve insulin resistance and its potential to prevent and treat diabetes.

#### 4.1.6 Puerarin

Puerarin, the major isoflavone glycoside isolated from the traditional Chinese medicine *Radix puerariae*, is widely used to treat diabetes and its complications. Mediated by the PI3K/AKT pathway, it is potent and directly protects β-cell survival and insulin secretion (Li et al., 2014). Moreover, the effect of 100 mg/kg puerarin for 4 days on C57BL/6 male mice with pancreatic β-cell toxic STZ significantly lowered blood glucose, reduced the incidence of diabetes, and directly protected the function and survival of pancreatic β-cell. Activating the PI3K/AKT pathway, considerably upregulated the p-AKT and Bcl-2 expression, and AKT phosphorylation was blocked when the LY 294002 inhibitor was involved. The mechanism of its action against lipid and glucose metabolism dysfunction was investigated after treatment with 300 mg/kg puerarin for 4 weeks in T2DM rats, fasting insulin, glycated hemoglobin, glucose tolerance and lipid profile were significantly normalized recovery and the expression level of PI3K, p-AKT, and p-FoxO1 was increased while PEPCK and G6Pase decreased (Liu et al., 2021). Further, its effect of suppressing gluconeogenesis and promoting glucose consumption by stimulating the PI3K/AKT pathway is also clarified in insulin resistance HepG2 cells (Shen et al., 2019; Liu et al., 2021).

#### 4.1.7 α-Methyl artoflavanocoumarin

α-Methyl artoflavanocoumarin (MAFC), a flavanocoumarin, is extracted from the heart part of *Juniperus chinensis* L. (Cupressaceae) (Orhan et al., 2011). As a novel PTP1B inhibitor, it inhibits PTP1B activity and upregulates the expression of PTP1B. Additionally, it also found that MAFC could significantly increase glucose uptake and dose-dependently enhance the protein levels of IRS-1, phosphorylated PI3K, and AKT, thus activating the IRS-1/PI3K/AKT signaling pathway in IR-HepG2 cells (Jung et al., 2017).

#### 4.1.8 Loureirin B

Loureirin B, a dihydrochalcone analog, extracted from Sanguis Draconis, which promotes insulin secretion and hypoglycemia (Fang et al., 2022). According to the research on loureirin B, 4 weeks of treatment with 45 mg/kg loureirin B in the HFD/STZ-induced T2DM mice model restored normalization of the liver index, insulin sensitivity, serum lipid content and liver glycogen content, and loureirin B at a concentration of 10–5 to 10–7 mol/L in IR-HepG2 cells affected the IRS1/PI3K/AKT/FoxO1 signaling pathway and regulated the expression of several essential genes and proteins in the pathway, thereby increasing glucose uptake and consumption, accelerating the conversion of glucose to glycogen, inhibiting hepatic gluconeogenesis, enhancing hepatic glycogen content and reducing insulin resistance (Ding et al., 2021).

#### 4.1.9 Fisetin

Fisetin, a flavonoid, is widely presented in natural plants. As an α-glucosidase inhibitor, it was identified to be a promising candidate for the treatment of T2DM (Shen et al., 2021). Fisetin supplementation could ameliorate hyperlipidemia and insulin resistance through regulating the IRS1^Tyr608^/AKT/GSK3β/FoxO1 signaling pathway, the expression of phosphorylated IRS1^Tyr608^, AKT, FoxO1 and GSK3β was markedly decreased by the intervention of fisetin in the kidneys of HFD-fed mice (Ge et al., 2019). Further, *in vitro* experiments have shown that fisetin increased the EGFR expression through IRS activating PI3K/AKT signaling pathway to alleviate hepatic IR (Li et al., 2023).

#### 4.1.10 Kaempferol

Kaempferol is a important dietary flavonoid that has been identified in many TCM (Fang et al., 2019). Kaempferol exhibits anti-diabetic effect in regulating hepatic gluconeogenesis and ameliorating fasting hyperglycemia and glucose intolerance through increasing AKT phosphorylation, oral administration of kaempferol improved insulin sensitivity and insulin resistance in HFD-fed mice (Alkhalidy et al., 2018). Moreover, kaempferol increased AKT phosphorylation in human SkM cells and in muscle of obese mice to stimulate glucose uptake and insulin resistance (Moore et al., 2023). Furthermore, kaempferol metabolites induce AKT and GSK3β phosphorylation and improve glucose metabolism (Fang et al., 2021).

#### 4.1.11 Quercetin

Quercetin is a naturally occurring flavonoid, ubiquitously present in fruits and vegetables (Fang et al., 2019; Dhanya, 2022). Quercetin has been shown to altering glucose homeostasis via glucose transporters and insulin signalling molecules, acts as potentiates IRS1, PI3K and AKT1 phosphorylation in brain of STZ-induced diabetic rats (Sandeep and Nandhini, 2017). In addition, a study showed that HFD-induced mice and PA-induced HepG2 cells treated with quercetin saw a enhancement alleviation of insulin resistance via the IRS-1/AKT/FoxO1 pathway, and stimulated expressions of p-IRS1, *p*-AKT and GLUT4 in liver (Cheng et al., 2021; Liu et al., 2022).

#### 4.1.12 Apigenin

Apigenin, a flavonoid, is widely distributed in folk medicines for diabetes treatment (Qin et al., 2016; Kashyap et al., 2021). Apigenin significantly increases glucose consumption and glycogen synthesis, suppresses the production of ROS and AGEs, and improves insulin resistance in IR-HepG2 cells, and elevated the level of protein expression of IRS-1, IRS-2, PI3K, and p-AKT is observed in IR-HepG2 cells (Miao et al., 2023). Therefore, the activation of the IRS-1/IRS-2/PI3K/AKT signaling pathway and regulation of its targets, including GLUT4 and GSK-3β, may play important roles in preventing diabetes (Fang et al., 2019; Miao et al., 2023).

#### 4.1.13 Poncirin

Poncirin, a natural flavonoid glycoside derivative present in the fruits of *Poncirus trifoliata*, possesses multiple biological activities (Li et al., 2022). Naringenin significantly increased glucose uptake and GLUT4 expression level via activating the IRS-1/PI3K/AKT/GSK-3 signaling pathway, and decreased the expression of PTP1B in IR-C2C12 skeletal muscle cells (Yousof Ali et al., 2020). Enhanced the phosphorylation of IRS-1, PI3K, GSK3β and AKT, and thus stimulated the glucose uptake and insulin sensitivity.

#### 4.1.14 Naringenin

Naringenin, a citrus flavonoid, has the ability to increase insulin secretion in the primary rat islets, protect β cell function and reverse glucose dysregulation in diabetic rats (Lin et al., 2023). Likewise, naringenin reduces lipid accumulation and insulin resistance through promoting AMPK phosphorylation level in liver of diabetic mice (Cai et al., 2023). Further, naringenin administration significantly altering glucose homeostasis, as well as significantly restored GLUT1 and GLUT3 expression, and increased the phosphorylated forms of IRS1, PI3K and AKT in a rat model of T2DM (Sandeep and Nandhini, 2017).

### 4.2 Polyphenols

**FIGURE 3:**
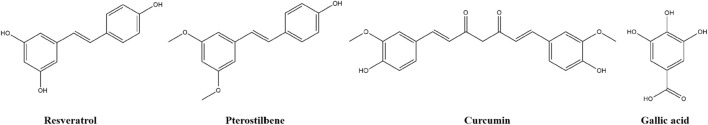
The chemical structures of four polyphenols.

Polyphenols are a group of chemicals formed by the combination of at least one aromatic ring with one or more hydroxyl functional groups attached to it, which are considered secondary metabolites and abundantly found in fruits, vegetables, and medicinal plants (Mirza-Aghazadeh-Attari et al., 2020). Meanwhile, Polyphenols were known to be instrumental in each of the vital processes of glucose metabolism. It prefers to arrest intestinal glucose absorption, increase pancreatic insulin secretion, enhance the capacity of muscle and adipocytes to utilize glucose and hinder glucose secretion by the liver (Shahwan et al., 2022), including resveratrol, pterostilbene, curcumin and gallic acid. The chemical structures of four polyphenols are shown in Figure 3.

#### 4.2.1 Resveratrol

Found in *Polygonum cuspidatum*, Resveratrol (3, 5, 4′-trihydroxystilbene, RSV) is a type of non-flavonoid polyphenolic with phytoalexin properties, which is particularly high in resveratrol and can be used as a TCM (Zhao et al., 2019). A series of studies have verified a broad spectrum activity of resveratrol in association with diabetes and its complications (Szkudelska and Szkudelski, 2010). In a metabolic action study in humans, it was shown that oral administration of 10 mg of RSV for 4 weeks significantly reduced insulin resistance and improved insulin sensitivity in humans and resulted in more efficient transduction of insulin signaling through the AKT pathway (Brasnyo et al., 2011). What counts is that researchers found that RSV restores the phosphorylation levels of AKT and PI3K in the liver of insulin resistance mice, which are involved in the insulin signaling pathway and were decreased by a high-fat diet (Hong et al., 2014; Sadi et al., 2015; Shu et al., 2020). Furthermore, RSV could also increase insulin secretion and produce a hypoglycemic effect in STZ-induced rats, KKAy mice, and HCF-fed rats via PI3K/AKT signaling pathway to enhance glucose uptake into skeletal muscles (Chi et al., 2007; Deng et al., 2008; Chen et al., 2012). In addition, the combination therapy of dapagliflozin with RSV has better glucose-lowering effects than the single SGLT2i therapy in T2D treatment, and the therapeutic effects of enhancing glucose production and inhibiting gluconeogenesis were also produced by modulating the PI3K/AKT pathway (Sun et al., 2021).

#### 4.2.2 Pterostilbene

Pterostilbene (trans-3, 5-dimethoxy-4′-hydroxystilbene; PTE), a polyphenol and a naturally occurring dimethylated analog, can be obtained from grapes and blueberries and be found in several TCM. Pterostilbene exhibits anti-diabetic effects both by normalizing the significant enzymes of glucose metabolism and regulating the insulin resistance signaling pathway (Pari and Satheesh, 2006). Furthermore, it can also reverse insulin resistance by decreasing the oxidative stress effect (Elango et al., 2016). The treatment of STZ-induced diabetic rats with pterostilbene at different concentrations (20, 40, and 80 mg/kg) for 8 weeks normalized the body weight, FBG, OGTT, serum lipid profile, and insulin levels in a dose-dependent manner (Sun et al., 2019). Moreover, the expression of PPARγ was increased, and the expression of PI3K and p-AKT was upregulated in adipose tissue of diabetic rats after treatments. The above results show that the mechanism of the anti-diabetic effect of pterostilbene in high-fat diet and STZ-induced diabetic rats may be related to the PI3K/AKT signaling pathway. A similar therapeutic effect of reversing insulin resistance was also demonstrated in HepG2 cells induced by palmitic acid *in vitro*. Meanwhile, pterostilbene can also regulate triglyceride accumulation and FFA metabolism and reduce oxidative damage to lipids via regulating the PI3K/AKT signaling pathway and the expression of genes coding for gluconeogenic enzymes (Malik et al., 2019).

#### 4.2.3 Curcumin

Curcumin (1, 7-bis (4-hydroxy-3-methoxyphenyl)-1, 6-heptadiene-3, 5-dione), a natural phenol found in *Curcuma longa* plants, has been shown to prevent hyperglycemia and hyperlipidemia as well as liver damage (Xia et al., 2020). Data indicated that administration of dietary curcumin reinstates PI3K and AKT levels in the liver of diet-induced obese mice and T2MD rats (Kim et al., 2019; Xia et al., 2020), ameliorates the tissue structure of the liver and pancreas and decreases blood glucose and lipid levels. It ameliorated insulin sensitivity via strengthening the PI3K/AKT/GSK3β signal pathway in high-glucose-induced IR HepG2 cells (Li et al., 2020). Additionally, the mechanistic basis of curcumin is a potential therapeutic strategy for the protection of pancreatic β-cells in T2DM, and it shows that curcumin protected MIN6 β-cells from palmitate-induced apoptosis by modulating the PI3K/AKT/FoxO1 signaling pathway and the mitochondrial survival pathway (Hao et al., 2015).

#### 4.2.4 Gallic acid

Gallic acid (3, 4, 5-trihydroxybenzoic acid), a naturally occurring phenolic acid, has been shown to possess anti-hyperglycemic and anti-diabetic activities in STZ-induced diabetic rats (Punithavathi et al., 2011; Punithavathi et al., 2011). *In vivo*, Gallic acid can improve insulin resistance in the liver, adipose, and skeletal muscle tissues of diabetic rats through translocation and activation of GLUT4 in the PI3K/AKT signaling pathway, and slightly upregulated the mRNA and protein expression levels of PPARγ (Gandhi et al., 2014). Its effects on hepatic glucose metabolism via regulating the PI3K/AKT pathway in HFD-induced diabetic rats were also confirmed (Huang et al., 2016). *In vitro*, the anti-diabetic effect of Gallic acid was found to protect against free fatty acid (FFA)-induced IR through the miR-1271/IRS-1/PI3K/AKT/FoxO1 pathway at dose of 50 μmol/L (Lee and Lee, 2021).

### 4.3 Alkaloid

**FIGURE 4:**
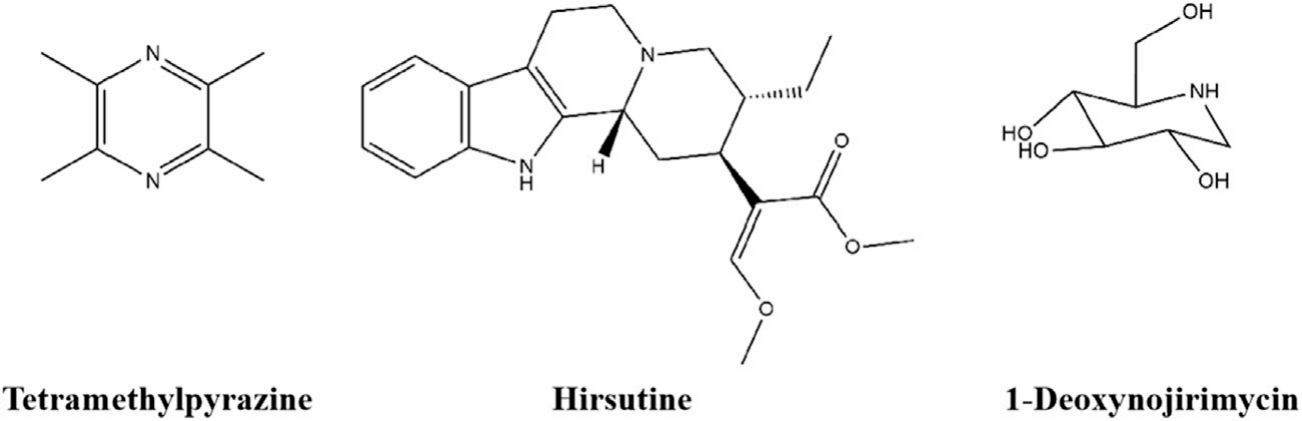
The chemical structures of three alkaloids.

Alkaloids are significant and excellent phytoconstituents found in medicinal plants and represent the beginning of the interesting potential for new approaches to the treatment of diabetes, and multiple *in vitro* and *in vivo* experiments have proven the relatively bright potential of alkaloids in the treatment of diabetes and its complications (Behl et al., 2022). Alkaloids such as tetramethylpyrazine, hirsutine, and 1-Deoxynojirimycin can regulate the balance between glycolipid metabolic and moderate insulin resistance. The chemical structures of three alkaloids are shown in Figure 4.

#### 4.3.1 Tetramethylpyrazine

Tetramethylpyrazine (TMP), also known as ligustrazine, is an alkaloid extracted from the plant Ligusticum chuanxiong with biological efficacy in improving glucose homeostasis and systemic insulin sensitivity (Xiang et al., 2020). Its anti-diabetic affection produced by reducing insulin resistance suppressing oxidative stress in HFD-STZ-induced T2D rats and STZ-NCT-induced T2D rats and suggested that the dose-dependent hypoglycemic activity and potential molecular mechanisms of the oral administration of TMP assessed by calculating the expression levels of phosphorylated PI3K and AKT proteins and mRNA in skeletal muscle, heart and adipose tissue of T2D rats (Rai et al., 2019; Rai et al., 2019). Therefore, it can be concluded that TMP improves insulin resistance and produces anti-diabetic activity by activating the PI3K/AKT signaling pathway.

#### 4.3.2 Hirsutine

Hirsutine is a potent drug-like indole alkaloid extracted from the *Uncaria rhynchophylla*. Hirsutine beneficially regulates glucose homeostasis, improving hepatic and cardiac IR in HFD-induced diabetic mice (Jiang et al., 2023). The ability of pharmacological anti-diabetic to promote glucose consumption and glycogen synthesis and to inhibit gluconeogenesis was also demonstrated adjuvantly in the IR model of HepG2 and H9c2 cells using high glucose and high insulin induction, and the mechanism of action was achieved through activation of PI3K/AKT/GSK3β signaling pathway (Hu et al., 2022).

#### 4.3.3 1-Deoxynojirimycin

1-Deoxynojirimycin, as an inhibitor of intestinal ɑ-glucosidase, the primary alkaloid isolated from mulberry leaves (*Morus alba* L.), has been reported as the critical practical bioactive material basis. Depending on previous research, the administration of purified 1-Deoxynojirimycin appeared to have antioxidant and anti-inflammatory roles in STZ-induced diabetic rats. It explains at least part of the mechanism by which it ameliorates blood glucose (Huang et al., 2014). Its therapeutic effect on hyperglycemia was evidenced by the reduction of blood glucose, serum insulin levels and HOMA-IR index while improving glucose tolerance and insulin sensitivity in skeletal muscle of db/db mice via activating insulin signaling PI3K/AKT pathway (Liu et al., 2015). Meanwhile, its mechanism of glucose homeostasis regulation effect in differentiated 3T3-L1 adipocytes by up-regulating the genes/proteins and mRNA expression of PI3K/AKT and AMPK signaling pathways from ADIPO to GLUT4 and from IR to GlUT4 (Li et al., 2019). Additionally, 1-Deoxynojirimycin supplementation appeared to improve muscle insulin resistance by modulating the IRS-1/PI3K/AKT pathway in the skeletal muscle of db/db mice (Kang et al., 2022).

### 4.4 Terpenoids

**FIGURE 5:**
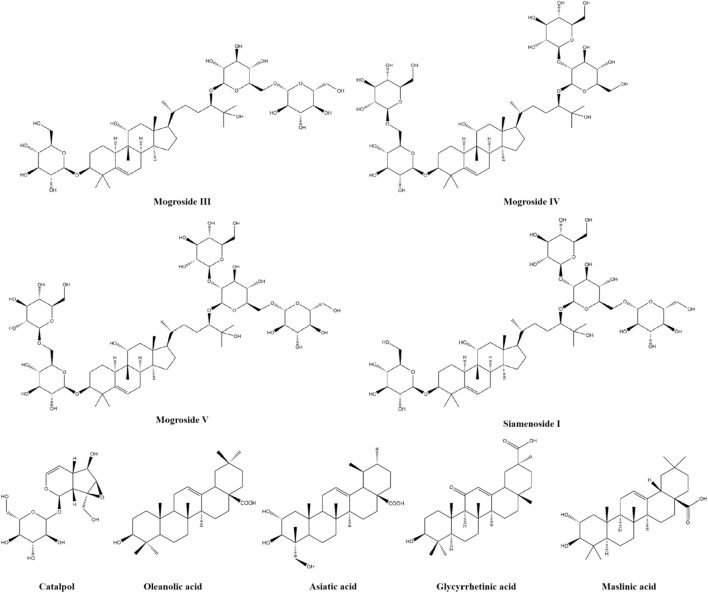
The chemical structures of nine terpenoids.

Terpenoids are widely distributed in nature among the most diverse phytochemicals, possessing a wide range of biological activities. Such compounds have been shown to modulate glycolipid metabolism and improve insulin resistance in terms of anti-diabetic activity (Szakiel et al., 2012). Terpenoids include siamenoside I, mogroside III, mogroside IV, mogroside V, catalpol, oleanolic acid, asiatic acid, glycyrrhetinic acid and maslinic acid. The chemical structures of nine terpenoids are presented in Figure 5.

#### 4.4.1 Mogroside

*Siraitia grosvenorii*, as a medicine food homology plant, possesses both nutritional and medicinal values. The fruit of S. *grosvenorii* is naturally enriched with sweetener compounds such as Mogroside III, Mogroside IV, Mogroside V, and Siamenoside I belong to triterpenoid constituents (Thakur et al., 2023). Researchers evaluated the hypoglycemic effect of four mogrosides, namely, Mogroside III, Mogroside IV, Mogroside V, and Siamenoside I. They reversed insulin resistance in IR-HepG2 cells by activating the PI3K signaling pathway, which can play a role in the regulation of glucose metabolism. Moreover, Mogroside V is the most significant curative effect compared with others. It has been found to alleviate glucose levels and insulin sensitivity in T2DM rats by regulating the PI3K/AKT signaling pathway (Liu et al., 2019).

#### 4.4.2 Catalpol

Catalpol, an iridoid glycoside with pharmacological benefits for the prevention of diabetes and diabetic complications, is mainly found in the roots of *Radix Rehmanniae* (Bai et al., 2019). The therapeutic effects of catalpol in controlling glycemic parameters in HFD/STZ-induced diabetic mice and its potential molecular mechanisms indicate that mRNA levels of IRS-1, PI3K, AKT2, and GLUT-4 in skeletal muscle were significantly improved by treatment (Yap et al., 2020). Meanwhile, catalpol improves insulin sensitivity and increases glucose uptake by enhancing MyoD/MyoG-mediated myogenesis. Moreover, in accordance with the research of *in vitro* and *in vivo* the mechanism of catalpol hypoglycemia in skeletal muscle involves modulation of the PI3K/AKT signaling pathway (Xu et al., 2018). Furthermore, the mechanism by which catalpol alleviates hepatic insulin resistance by regulating the expression of the PI3K/AKT pathway and its downstream glucose metabolism-related proteins has been demonstrated in T2D mice and IR-HepG2 cells models (Yan et al., 2018).

#### 4.4.3 Oleanolic acid

Oleanolic acid (3β-hydroxyolean-12-en-28-oic acid), a glycogen phosphorylase (GP) inhibitor, is a naturally occurring pentacyclic triterpene widely found in a range of foods and TCM and has enormous potential in hypoglycemic effects. Oleanolic acid could improve blood glucose and insulin homeostasis by enhancing the phosphorylation of ATK and AMPK in the liver of db/db diabetic mice and significantly improve hepatic gluconeogenesis and hepatic pathological changes (Wang et al., 2015). Further, additional research data manifest that the sustained modification of glucose homeostasis by oleanolic acid is due, at least in part, to the repression of AKT/FoxO1 axis-mediated gluconeogenesis in liver (Zeng et al., 2012). Meanwhile, oleanolic acid attenuated adipose tissue insulin resistance induced by fructose over-consumption in rats via IRS-1/PI3K/AKT signaling pathway (Li et al., 2014).

#### 4.4.4 Asiatic acid

Asiatic acid is a natural pentacyclic triterpenoid derived from *Centella Asiatica* (L.) Urban and exhibited potent hepatoprotective biological function. Oral administration of asiatic acid to STZ-induced diabetic rats restored the key carbohydrate-metabolizing and lipid metabolic enzymes and lipid peroxidation products to nearly normal levels. Reliable research data found it positively lowered blood sugar, lipid, and lipid peroxidation (Ramachandran and Saravanan, 2013; Ramachandran and Saravanan, 2013; Ramachandran and Saravanan, 2015). Additionally, it also found that its hypoglycemic effect may activate the PI3K/AKT signaling pathway to significantly improve glucose uptake and insulin resistance in skeletal muscle tissue of STZ-induced diabetic rats and improve glucose homeostasis (Ramachandran and Saravanan, 2015). Its anti-diabetic effects have also been demonstrated in T2DM (db/db) mouse models, and it promoted glycogen synthesis by activating PI3K/AKT/GSK-3β signaling pathway in liver tissue (Sun et al., 2017).

#### 4.4.5 Glycyrrhetinic acid

Glycyrrhetinic acid, a triterpenoid, is one of the main bioactive components in licorice (Tan et al., 2022). Glycyrrhetinic acid prevented hyperglycemia and hyperlipidemia in STZ-induced diabetic rats and improved to normalcy (Kalaiarasi et al., 2009). In addition, glycyrrhetinic acid elicits its anti-diabetic activity mainly through regulating the PI3K/AKT/GSK-3β signaling pathway (Yang et al., 2020; Tan et al., 2022; Meng et al., 2023). Moreover, glycyrrhetinic acid was found to decrease the activation of the phosphorylation of IRS1ser307 and increased the phosphorylation of AKTser473 and GSK-3βser9 in IR-HepG2 cells, thus improving insulin-response pathway and glucose consumption levels (Zhang et al., 2019; Wang et al., 2023).

#### 4.4.6 Maslinic acid

Maslinic acid is a pentacyclic triterpene acid that possesses a variety of biological activities, and has been shown to regulate glycogen metabolism in HFD-induced diabetic mice as a glycogen phosphorylase inhibitor (Liu et al., 2014; Yan et al., 2023). In addition, maslinic acid intervention on IR-HepG2 cells elevated the phosphorylation levels of AKT and GSK-3β, and PI3K inhibitor blocked the phosphorylation of AKT^Ser473^, thus proving its potential to regulate glycogen metabolism (Liu et al., 2014). Further, identified that maslinic acid has potent anti-adipogenic effects to target adipocyte function and prevent obesity, and target activation of PI3K/AKT signaling pathway (Savova et al., 2021).

### 4.5 Quinones

**FIGURE 6:**
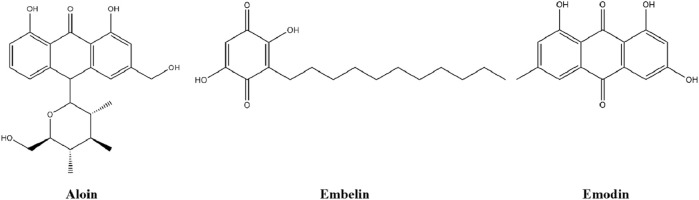
The chemical structures of three quinones.

Quinones are a class of aromatic dicarbonyl that are widely found in nature and possess a wide range of biological activities, of which anthraquinone is the largest naturally occurring quinone, such as aloin, embelin and emodin (Yang et al., 2020). The chemical structures of three quinones are shown in Figure 6.

#### 4.5.1 Aloin

Aloin, isolated from the leaf secretion of *Aloe vera* (L.) Burm. f., belonging to anthraquinone compounds (Jiang et al., 2018). Both *in vivo* and *in vitro* studies of aloin have shown its hypoglycemic effects (Zhang et al., 2020), with *in vivo* studies demonstrating that aloin improves glucose tolerance and fasting serum insulin activity in T2D mice and has hepatoprotective effect, which is mediated by activation of the IRS1/PI3K/AKT pathway (Cui et al., 2014), and *in vitro* studies demonstrating that aloin markedly improves glucose consumption and stimulates the activity of key enzymes of glucose metabolism in IR-HepG2 cells (Zhong et al., 2022).

#### 4.5.2 Embelin

Embelin (2, 5-dihydroxy-3-undecyl-1, 4-benzoquinone), a naturally occurring alkyl-substituted hydroxyl benzoquinone, isolated from *Embelia ribes* Burm, which has been extensively evaluated for its anti-diabetic activity (Durg et al., 2017). Embelin treatment of HFD-STZ-induced T2DM rats shows that it regulates glucose uptake by regulating GLUT4 transposition and activation in epididymal adipose tissue mediated by insulin-dependent PI3K/AKT pathway, manifesting that it plays a positive role in improving adipose tissue insulin sensitivity, enhancing blood glucose control, protecting β cells from damage and maintaining adipose tissue glucose homeostasis in animal models (Gandhi et al., 2013).

#### 4.5.3 Emodin

Emodin, an anthraquinone, was characterized being an active agent in lowering blood lipids and modulating glucose utilization (Yu et al., 2023). It was demonstrated that emodin improves hepatic glucose utilization, muscle and fat glucose uptake by targeting the IRS/PI3K/AKT/FoxO1 pathway, resulting in enhancing insulin sensitivity and resistance, the protein expression of IRS1, PI3K, and p-AKT ser473 in hepatic, muscle, and adipose tissue of diabetic mice was upregulated (Xuezheng et al., 2018).

### 4.6 Saponins

**FIGURE 7:**
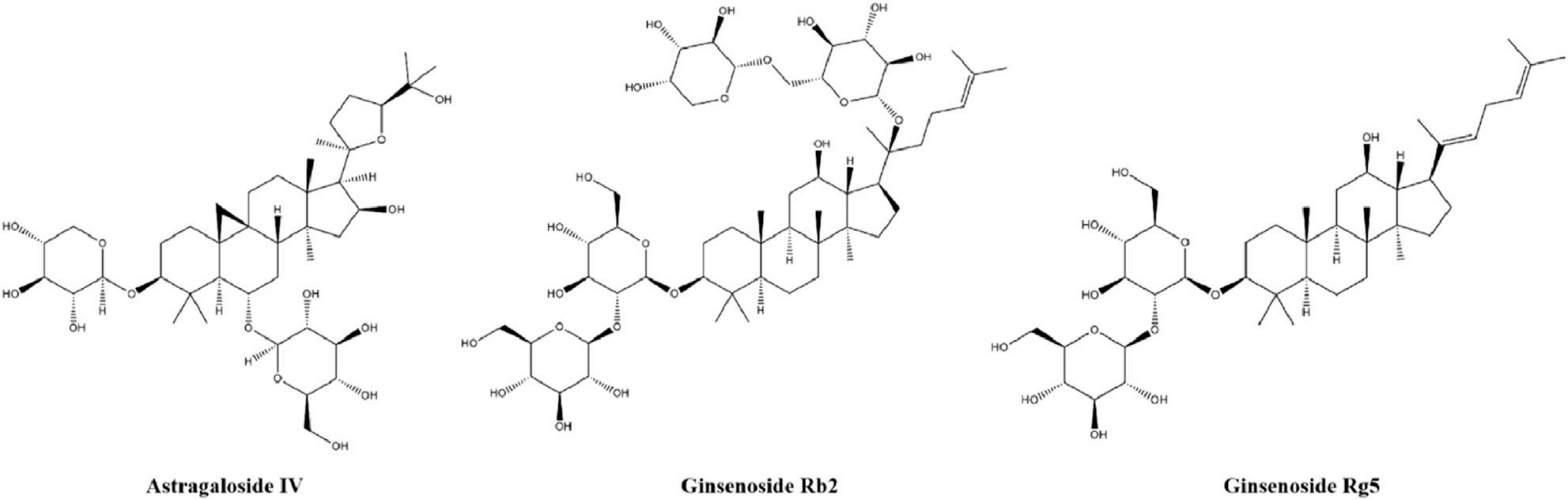
The chemical structures of three saponins.

Saponins, surface-active glycosides widely found in TCM, usually consist of a structure linking both a glycoside and a hydrophobic glycosidic ligand (saponin element), which in nature can be triterpenoids or steroids in nature (Elekofehinti, 2015). Their anti-diabetic properties have been demonstrated by their reported activities of regulating glucose-lipid metabolic homeostasis, promoting insulin secretion and enhancing insulin sensitivity as shown *in vivo* and *in vitro* models of insulin resistance. Astragaloside IV, ginsenoside Rb2 and ginsenoside Rg5 are examples of saponins. The chemical structures of three saponins are shown in Figure 7.

#### 4.6.1 Astragaloside IV

Astragaloside IV, a glycoside of cyclobutane-type triterpene obtained from *Astragalus membranaceus*, has the effect of preventing diabetes by inducing a decrease in blood glucose concentration and an increase in plasma insulin levels (Yu et al., 2006). It can reduce blood glucose levels in HFD-STZ-induced diabetic mice, glycogen phosphorylase (GP) and glucose-6-phosphatase (G6Pase), two glucose-regulated enzymes, were inhibited to improve glucose metabolism in the liver (Lv et al., 2010). Additionally, it inhibits lipolysis and reduces hepatic glucose production in HFD-fed mice through AKT-dependent PDE3b expression (Du et al., 2018). Its mechanism of action is reducing insulin resistance in adipocytes by regulating CTRP3 and PI3K/AKT signaling (Zhang et al., 2022).

#### 4.6.2 Ginsenosides

Ginsenosides, obtained from ginseng, have been demonstrated to possess anti-diabetic activity, such as Ginsenoside Rb2, Ginsenoside Rg5, etc. Ginsenoside Rg5 may be a potential natural product in the treatment of T2DM for the first time, which can remarkably improve glucose and lipid metabolism, increase insulin secretion, and protect damaged tissues in T2D mice. Further, it improves liver insulin resistance in db/db mice and alleviates T2DM by regulating IRS1/PI3K/AKT/GSK3β signaling pathway (Wei et al., 2020). Ginsenoside Rb2 can jointly improve insulin resistance of 3T3-L1 adipocytes and DIO mice by regulating various pathways such as PI3K/AKT, MAPK and NF-κB, showing various therapeutic effects such as upregulation of inflammatory factors, reduction of fat accumulation and improvement of glucose metabolism (Dai et al., 2018).

### 4.7 Others

**FIGURE 8:**
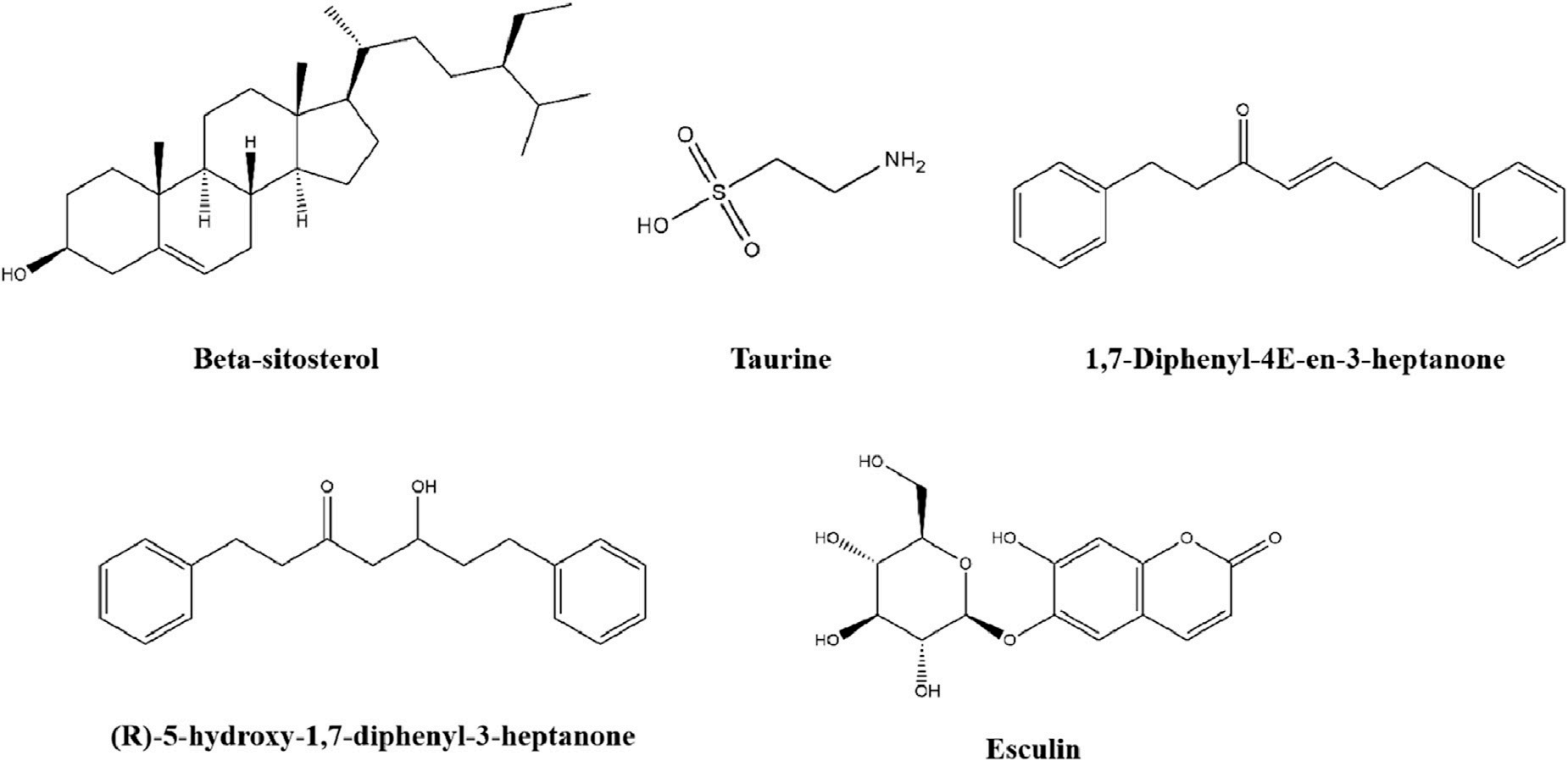
The chemical structures of five others.

There are some other metabolites of TCM, including beta-sitosterol, taurine, 1, 7-Diphenyl-4E-en-3-heptanone (DPH5), (R)-5-hydroxy-1, 7-diphenyl-3-heptanone (DPHC) and esculin, are also used to treat T2DM. The chemical structures are presented in Figure 8.

#### 4.7.1 Beta-sitosterol

Previous studies have found that the serum insulin level in patients with type 2 diabetes is negatively correlated with the plasma non-cholesterol sterols concentration, which suggests that supplementation of phytosterols may have beneficial effects on lowering blood glucose levels and preventing T2DM (Šmahelová et al., 2005). Beta-sitosterol is one of the most common phytosterols, widely distributed in many plants and often found in herbal formulations to treat hypercholesterolemia, coronary artery disease, and prostatic hyperplasia (Ponnulakshmi et al., 2019). Studies have shown that it can protect the expression of insulin signaling molecules in adipose tissue and skeletal muscle of rats with T2DM induced by a high-fat diet and sucrose and improve blood glucose metabolism by enhancing the expression level of insulin receptor (IR) and GLUT4 and regulating the IRS-1/AKT mediated signaling pathway (Babu et al., 2020). It was suggested that beta-sitosterol has anti-diabetic potential. Furthermore, it affected glucose transport and lipid mobilization in primary preadipocytes from male rats by activating and regulating the PI3K/AKT signaling pathway and GLUT4 expression level (Chai et al., 2011). The anti-diabetic potential in a skeletal muscle model established using L6 myotube cells and found that it promoted glucose transport and glucose uptake in L6 myotube cells by activating IRS-1/PI3K/AKT signaling pathway and improving GLUT4 expression level (Sujatha et al., 2010). The above results manifest that insulin-like property is one of the mechanisms of improving insulin resistance *in vitro*.

#### 4.7.2 Taurine

Taurine, obtained from *Bos taurus* domesticus Gmelin, is a sulfur-containing amino acid (Rashid et al., 2013). It can regulate a variety of the body’s normal physiological activities, such as participating in the beta cell function, regulating insulin signaling pathways and glucolipid metabolism of the liver (Das et al., 2012; Batista et al., 2013; Tang et al., 2019; Zhao et al., 2019). Taurine could improve insulin resistance by activating the PI3K/AKT/GLUT4 pathway in HFD/STZ-induced T2DM rats and PA-induced IR-HepG2 and the regulatory effects of taurine on the insulin signaling pathway in the liver, the target organ of insulin. Moreover, its potential to prevent T2DM was evaluated *in vitro* and *in vivo* (Chen et al., 2021).

#### 4.7.3 Diarylheptanoid

1,7-Diphenyl-4E-en-3-heptanone (DPH5) and (R)-5-hydroxy-1,7-diphenyl-3-heptanone (DPHC) are diarylheptanoid present in the rhizome of *Alpinia officinarum* Hance (Xin et al., 2017; Abubakar et al., 2018). These metabolites are considered the most active bioactive metabolites extracted from this plant and have favorable hypoglycemic effects. DPH5 could promote glucose uptake and consumption of IR-HepG2 cells, accelerate glucose utilization, and improve insulin resistance and insulin sensitivity by regulating PI3K/AKT-GSK3β signaling pathway and increasing the expression of GLUT4 and GSK3β proteins (Zhang et al., 2022). Additionally, DPHC could regulate glucose metabolism and hypoglycemic activity well in both db/db mouse models and *in vitro* high-glucose induced IR-HepG2 cells, and its mechanism improves insulin resistance by regulating IRS1/PI3K/AKT/GLUT4 signaling pathway, showing potential for T2DM treatment (Zhang et al., 2021).

#### 4.7.4 Esculin

Esculin, a plant derived natural coumarin extracted from *Cortex fraxini*, is considered to exert multiple anti-diabetic properties (Naseem et al., 2023). Esculin amelioration of unhealthy AT remodeling was also proven for the first time as a novel therapeutic strategy for obesity-induced IR. Intervention with esculin could enhance insulin sensitivity and improve adipose tissue remodeling in obese IR C57BL/6J mice (Ghosh et al., 2022). In PA-treated adipocytes, esculin could promoted glucose uptake through increasing the enhancement of GLUT4 translocation and the expression of p-PI3K p85^Tyr467^, p-AKT^Ser473^, and p-IRS1^Ser307^ (Yang et al., 2024). In addition, esculin restored blood glucose level and glucose tolerance in STZ-induced diabetic mice and dexamethasone-induced diabetic mice, and enhance the phosphorylation of AKT in C2C12 myotubes, indicating a potential for the improvement of insulin resistance (Kang et al., 2014; Mo et al., 2019).

## 5 Future directions and perspective

### 5.1 The role of the PI3K/AKT signaling pathway for hypoglycemic effect in T2DM

As a chronic metabolic disease, the pathogenesis of T2DM has not yet been fully elucidated. The available therapeutic drugs can only alleviate the symptoms of the disease, failing to achieve a completely curative effect, and tend to be accompanied by a multitude of adverse effects. According to extensive research, the PI3K/AKT signaling pathway holds a significant advantage for hypoglycemic effect in T2DM, as it can effectively ameliorate insulin resistance in peripheral target organs of insulin. To some extent, it compensates for the shortcomings of the commonly used anti-diabetes drug, metformin (an AMPK agonist), in clinical treatments. However, it is worth noting that the role of the PI3K/AKT signaling pathway is quite extensive, having connections with numerous diseases, This review found that extensive preclinical studies have demonstrated that activation of the PI3K/AKT signaling pathway can significantly improve various abnormal indicators of T2DM *in vivo* and *in vivo*, and regulating glucose and lipid metabolism levels in insulin target organs and target cells, to some extent, improving insulin resistance and exerting hypoglycemic effects. Although there is no recent progress in clinical studies on the metabolites of TCM as the signaling pathway modulators, the performance of the metabolites of TCM in basic research provides a valuable theoretical basis for entering clinical studies in the future. However, it is worth noting that the PI3K/AKT signaling pathway has quite a wide range of roles and has been associated with numerous diseases, and thus further insight is needed as to whether activation of this pathway directly benefits T2DM without triggering other effects. Moreover, regulating the expression of the PI3K/AKT pathway and its downstream effector proteins is far from sufficient, and the precise targets and mechanisms of diabetes treatment need to be further elucidated in detail.

Moreover, as the TCM exert an immeasurable potential in the treatment of chronic diseases and due to the characteristics of many TCM as homologous with food and medicine, the metabolites of TCM also tend to have lower and fewer side effects and exhibit higher clinical efficacy, attracting considerable attention from relevant academic researchers. Upon these studies, we found that these active metabolites could not only improve glycogen synthesis and glucose uptake, but also inhibit gluconeogenesis through the PI3K/AKT signaling pathway, so that they could improve insulin resistance and exert hypoglycemic effects. In addition, a lot of metabolites of TCM could regulate glucose metabolism and play a comprehensive regulatory role in treatment of T2DM. Therefore, we strongly believe that the TCM are on the verge of breaking new ground in the treatment of T2DM.

### 5.2 Characters of metabolites of traditional Chinese medicine in regulating PI3K/AKT signaling pathway

1) Flavonoids, polyphenols and terpenoids

On the basis of compound categorization, researchers have studied the metabolites of flavonoids, polyphenols and terpenoids in metabolites of TCM more extensively compared to alkaloids, quinones and saponins. The effects of flavonoids, polyphenols and terpenoids on regulating the expression levels of glycogen synthesis, gluconeogenesis and glucose uptake-related proteins mediated by the PI3K/AKT signaling pathway were explored more deeply and roundly.

Flavonoid demonstrate significant activity and simpler structures, giving them a slight advantage in terms of druglikeness and bioavailability. It is also possible to further modify and optimize their structures and develop new dosage forms to improve the pharmacokinetic properties of these metabolites of TCM so that more efficient drugs with fewer toxic side effects can be designed.

2) Quinones and saponins

Quinones and saponins have been shown to activate the PI3K/AKT signaling pathway in the preclinical stage, and p-PI3K and p-AKT showed an increase in protein and/or mRNA expression levels upon drug action, but the effects on the downstream proteins of the pathway have been less studied, and need to be further explored in depth.

3) Alkaloids

It is noteworthy that probably the researchers took into account that alkaloids often contain toxicity, so the number of alkaloid with hypoglycemic properties in our collection was relatively small. However, they showed different degrees of enhancement on the PI3K/AKT signaling pathway and its downstream-mediated effector proteins, and all of them demonstrated high improvement of insulin resistance and hypoglycemic bioactivities *in vivo* and *in vivo* studies. Therefore, alkaloids metabolites of TCM have great development prospect in treatment of T2DM.

Furthermore, in preclinical studies, metabolites of TCM have exhibited considerable potential activity. However, given the complex structures of some metabolites, their corresponding druglikeness, bioavailability and toxicity require further scrutiny, as do their synthetic processes examples, including terpenoids, quinones and saponins.

Relevant illustrations of the conversion of the potentially metabolites of TCM from preclinical to clinical studies demonstrate the hypoglycemic activity of stevioside and rebaudioside A extracted from *Stevia rebaudiana* Bertoni in human subjects (Mohammed et al., 2022). For instance, in an acute, paired cross-over study, supplementation of the test meal with 1 g of stevioside resulted in a reduction in postprandial blood glucose, with stevioside reducing the incremental area under the glucose-response curve by 18% compared to the control group (Gregersen et al., 2004; Barriocanal et al., 2008). Furthermore, the chronic intake of 1,000 mg/d of rebaudioside A was well-tolerated without hypoglycemia or alteration of blood pressure in patients with type 2 diabetes mellitus (Maki et al., 2008). Additionally, supplementation with the natural polyphenol resveratrol 150 mg/day for 30 days inhibited postprandial glucagon responses in patients with obesity and did not affect postprandial incretin hormone responses (Knop et al., 2013). Despite the abundance of metabolites of TCM that have demonstrated better activity in preclinical studies, their corresponding clinical studies are relatively scarce. Moreover, direct studies on the effects of metabolites of TCM on the PI3K/AKT pathway in T2DM are not available so far, but a large number of basic studies provide a solid foundation for future clinical studies. Nonetheless, the clinical application of TCM as modulators of the PI3K/AKT pathway in the context of T2DM still needs to be further developed and explored.

## 6 Conclusion

In conclusion, the metabolites of TCM operates on both *in vivo* and *in vitro* models, activating the PI3K/AKT signaling pathway, enhancing glycogen synthesis and glucose uptake, and inhibiting gluconeogenesis in insulin-targeted organs, thus ameliorating insulin resistance and exerting hypoglycemic effects. Research on metabolites of traditional Chinese medicine for treating T2DM predominantly remain at the primary stage, however, these studies lay a robust foundation for future clinical investigations.
